# Current Analytical Methods and Recent Advances in Histamine Analysis in Fish and Fish Products: A Systematic Review

**DOI:** 10.3390/foods15152590

**Published:** 2026-07-23

**Authors:** Miguel Henares, Doaa Abouelenein, Lene Duedahl-Olesen, Maribel Gómez-Gómez, Amadeu Griol, Isabel Fernández-Segovia, Ana Fuentes, José Manuel Barat

**Affiliations:** 1 University Institute of Food Engineering-FoodUPV, Universitat Politècnica de València, Camino de Vera s/n, 46022 Valencia, Spain; t82heolm@uco.es (M.H.); isferse1@tal.upv.es (I.F.-S.); jmbarat@tal.upv.es (J.M.B.); 2 Research Group for Analytical Food Chemistry, National Food Institute, Technical University of Denmark (DTU), Kemitorvet 202, DK-2800 Lyngby, Denmark; ddaab@food.dtu.dk (D.A.); ldup@food.dtu.dk (L.D.-O.); 3 Nanophotonics Technology Center, Universitat Politècnica de València, Camino de Vera s/n, 46022 Valencia, Spain; migmegme@ntc.upv.es (M.G.-G.); agriol@upvnet.upv.es (A.G.)

**Keywords:** histamine, analytical methods, food analysis, fish, fish products

## Abstract

Histamine (HIS) in food products can cause poisoning or intolerance reactions, which are usually associated with fish and seafood consumption. Therefore, the detection and quantification of histamine in fish and fish products are important for public health protection. This article provides an overview on the current methodologies and recent advances in histamine detection and quantification applied to fishery products. Among the evaluated studies, chromatographic techniques, especially HPLC, are the most common due to their good performance. However, chromatographic techniques present disadvantages, such as complex sample preparation and use of organic solvents, and attempts have been made to solve them with spectroscopic and enzymatic methodologies, and sensors models. Sensor-based methods (biosensors, electrochemical sensors, optical sensors) allow real-time or near-real-time detection of histamine, making them highly valuable for on-site quality control. The incorporation of selective recognition elements, such as enzymes, aptamers, or molecularly imprinted polymers (MIPs), significantly enhances the selectivity and reduces detection and quantification limits. However, challenges related to stability, interference, and lack of standardisation can limit their ability to fully replace traditional laboratory methods. Future research should therefore focus on developing robust, cost-effective, and standardised detection platforms capable of combining the accuracy of chromatographic methods with the speed and practicality of modern sensors, ultimately strengthening food quality control and reducing the risk of histamine-related intoxications worldwide.

## 1. Introduction

Histamine (HIS) is a biogenic amine (BA) with a heterocyclic structure [1] that is naturally present in the human body and is involved in essential physiological and metabolic functions. HIS can also appear in some foods and drinks, particularly in fermented products like cheese, beer and red wine, and especially in fish or fish products containing high concentrations of its precursor amino acid, histidine. During storage or processing, histidine can be transformed into HIS through a microbial decarboxylation reaction. At low levels, HIS is usually harmless because it is detoxified by conjugation or oxidation in the human intestinal tract. However, ingestion of an excessive amount of HIS causes severe symptomatology in what is known as “histamine poisoning” or “scombroid fish poisoning”. Symptoms are skin rashes, headache, nausea, and diarrhoea, among other pathologies, which may result in toxicity and metabolic disturbance and can be harmful for the nervous and cardiovascular systems [2]. However, certain individuals’ inability to metabolise HIS in the intestine can explain some uncertainties historically associated with HIS intoxication. In this sense, HIS intolerance is generally associated with reduced activity of histamine-degrading enzymes, rather than excessive histamine formation in the food itself.

In fish and fish products, the level of HIS is considered an indicator of hygiene quality. Histamine accumulation is strongly influenced by storage temperature, cold-chain interruptions, and exposure to inadequate time-temperature conditions; consequently, rapid analysis methods are becoming increasingly valuable for timely monitoring throughout the stages of processing, distribution, retail, and consumer storage. The Food and Drug Administration (FDA) establishes that fish is unsafe for human consumption if it contains 200 mg /kg of HIS, and HIS levels 35 mg/kg or greater indicate significant decomposition and mishandling of the fish [3]. In the EU, accepted HIS levels vary based on the type of product and fish species, focusing on fish species associated with high histidine content (e.g., *Scombridae*, *Clupeidae*, *Engraulidae*, *Coryphaenidae*, *Pomatomidae*, *Scomberesocidae*), for fresh fish [4]. For fishery products, compliance is based on a nine-unit sampling plan rather than a single cutoff: the mean histamine content must not exceed 100 mg/kg, no more than two units may fall between 100 and 200 mg/kg, and no unit may exceed 200 mg/kg. For fishery products that have undergone enzyme maturation in brine, the corresponding EU criteria are 200 mg/kg for the mean and 400 mg/kg for the maximum individual unit under the same *n* = 9, *c* = 2 sampling plan, whereas fish sauce produced by fermentation is subject to a single-sample limit of 400 mg/kg because histamine is expected to be more uniformly distributed in that product [4]. Although no adverse health effects from the consumption of food containing below 50 mg of HIS per meal for healthy individuals have been observed, ingestion of low HIS levels (between 5 and 10 mg HIS) by HIS-sensitive individuals may cause an adverse reaction [5].

For this reason, high-sensitivity techniques to detect HIS within a wide range of complex matrices are necessary for public health protection. The challenge of HIS analysis is often the complexity and variability of food matrix characteristics and the presence of potentially interfering compounds, including other BAs like tyramine, putrescine or cadaverine. Current analytical methods generally include amine extraction and derivatisation, followed by chromatographic separation and spectroscopic quantification. Analytical methods based on reverse-phase High Performance Liquid Chromatography (HPLC) separation, followed by UV-Vis, fluorescence (FL) or mass spectrometry (MS) detection [6], are commonly employed for HIS quantification. ISO Standard 19343:2017 specifies an HPLC method to analyse HIS in fish and fishery products [7]. According to the ISO method, BAs are extracted from a sample with perchloric acid, followed by precolumn derivatisation using dansyl-chloride (DNS-Cl). Then, BAs are separated by HPLC, followed by UV detection. In addition, capillary electrophoresis with different detectors, and Gas Chromatography (GC) coupled to an MS detector, have been previously applied for the separation and detection of BAs in different food products [6]. The AOAC 977.13 method for fish recommends a fluorometric assay with HIS extraction in methanol followed by ion-exchange column separation and postcolumn derivatisation before FL detection [8]. Some enzymatic methods exist like the enzyme-linked immunosorbent assay (ELISA), a colorimetric assay [9], as well as histamine-producing bacteria-detecting methods based on histidine decarboxylase. Despite the high sensitivity of these techniques, some authors consider them to be expensive, requiring sophisticated instrumentation and qualified personnel, as well as specific sample preparation steps [10,11]. These limitations have been addressed through the development of methods based on sensor models. Recent advances in HIS sensors have been driven by progress in nanotechnology, such as the use of graphene and gold nanoparticles to increase sensitivity; biotechnology, such as the use of stable aptamers and synthetic MIPs to increase accuracy; and digital integration, such as smartphone-based analysis and artificial intelligence. Sensors can now be considered less expensive, rapid, cost-effective techniques that can be used on-site. However, challenges remain, such as a higher limit of detection (LOD) and low sensitivity and selectivity for individual amines and total bile acids [12]. Therefore, this work aims to review the current available analytical methods for HIS analysis in fish and fishery products, and to compile recent advances in current analytical techniques for quantitative HIS determination, focusing on novel developments in the food sensors models and future trends. Unlike previous reviews focused primarily on conventional or novel detection technologies, this work integrates a comparison of chromatographic methods, spectroscopic approaches, electrochemical sensors, biosensors, and emerging portable formats, covering aspects ranging from sample preparation and analytical performance to the potential for routine or on-site monitoring of the quality and safety of fish and fishery products. This review provides a unique, matrix-specific evaluation of traditional and emerging detection strategies. The most pertinent practical aspects of all applied methodologies are assessed, including sample preparation requirements, limits of detection and quantitation, precision, recovery, and analysis time.

## 2. Methods

The present systematic review was carried out following PRISMA (Preferred Reporting Items for Systematic Reviews and Meta-analyses) guidelines [13] to identify the most relevant articles that focus on traditional and novel methods for HIS determination in fish and fish products. The completed PRISMA checklist is provided as a Supplementary Material.

### 2.1. Search Methodology

The literature search was carried out using three electronic databases: Web of Science, PubMed and Scopus. The selected keywords were “Histamine analysis” and “Fish OR Fish Products”. Articles’ years of publication started from 2015, and the date that every website database was searched was September 2025.

The resulting articles were exported to EndNote reference manager.

### 2.2. Study Selection Criteria

The criteria employed for the publication screening are shown in Table 1.

### 2.3. Articles Selection Process

Duplicates were firstly removed by using EndNote document manager. Next a second removal of duplicates was manually done. Then, the titles and abstracts of the obtained articles were scanned independently by two review authors, as shown in Table 1. Disagreements were resolved by a third author by later discussion. If these publications satisfied the selection criteria and met the review requirements, full texts were reviewed to identify potential works.

Eligible articles were then assessed. The information evaluated from the selected studies included the analytical method used in the protocol, food matrix, sample treatment, goodness of the method validation, range of concentrations, time of analysis, specific parameters of the analytical method, as well as advantages and disadvantages of the HIS determination technique. The collected articles were classified according to the technique used for the HIS analysis into the following categories: chromatographic (HPLC and non-HPLC methods), spectroscopic, enzymatic, electrochemical, and biosensor-based methods.

## 3. Results and Discussion

### 3.1. Bibliographic Search Results

**Figure 1:**
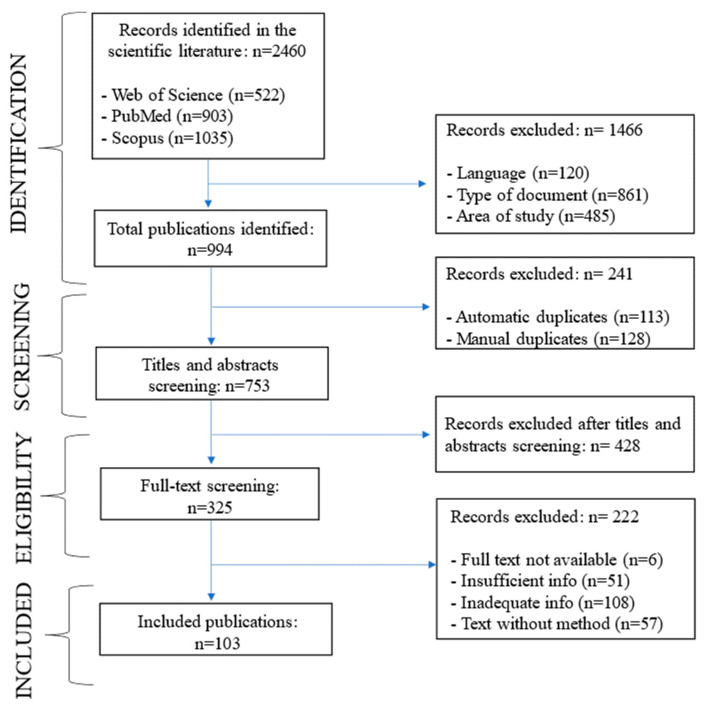
PRISMA Flow Diagram for search and screening processes.

From the three databases, 2460 articles were initially identified. After applying all the exclusion criteria, the number of articles was reduced to 994 (120 were removed due to language, 861 because of type of document, and 485 for not matching the study area). Removing duplicates left 753 eligible articles to be screened for title, abstract and full text. Eligibility criteria were established a priori following the PRISMA guidelines to ensure a transparent and reproducible study selection process. After a thorough evaluation of the inclusion and exclusion criteria (Table 1), a total of 103 full-text articles were selected (Figure 1). Only scientific articles published between 2015 and 2025 were included in order to include recent developments in histamine (HIS) analytical methodologies. Studies were required to focus on histamine (HIS) analysis in fish or fish products and to provide a clear description of the analytical methodology and/or method validation, with results reported specifically for HIS. Publications not directly related to the objectives of the review, including clinical studies, books, conference proceedings, randomised controlled trials, studies focused on microbiology or toxicology, or articles lacking methodological details on histamine analysis, were excluded. Other studies referenced in the included articles (which did not appear in the initial search) were added for their relevance to the research. This was specifically the case of the studies in which other food matrices besides fish and fishery products were also incorporated, as they describe methodological developments, optimisation strategies, or validation approaches that could be directly applicable to the determination of histamine in fish and fishery products.

### 3.2. Chromatographic Methods

#### 3.2.1. HPLC Methods

Table 2 summarises the different HPLC methods applied to HIS determination and their performance parameters. Information on the food matrix in which these protocols are applied and sample preparation protocols are also described. An extended description of these chromatographic protocols, including details of the employed column, mobile phase composition, flow rate, temperature, injection volume and detection parameters of, is shown in Table S1 of the Supplementary Material.

HPLC is the most widely used technique for analysing BAs in food and biological samples. Sample preparation for HIS chromatographic analysis is similar in most of the revised protocols, including extraction in acidic medium using inorganic acids at a low concentration like HClO_4_ [14,15], HCl [16,17], HNO_3_ [18], and trichloroacetic acid (TCA) [19], among others. To increase the extraction yield, several techniques have been applied. The most common technique was ultrasonic bath extraction [20,21], usually accompanied by heating [17]. Due to some samples’ complexity, it is necessary to include cleaning and purifying steps to remove possible interferences. For this purpose, solid-phase extraction (SPE) using a C18 cartridge [22], liquid/solid-phase transition microextraction (LSPTME) [23], QuEChERS [21], dispersive liquid–liquid micro-extraction (dLLME) [24] or magnetic (CoFe_2_O_4_) solid-phase extraction (MSPE) [25] were employed. Carrez solution was also utilised to precipitate remaining proteins in the extract [26].

The HPLC technique allows the separation of HIS and other BAs from a mixture eluted in mobile phases, such as water:acetonitrile (ACN), phosphate buffer:ACN or methanol:water through a chromatographic column of variable dimensions containing a stationary phase. After separation, analytes can be identified and quantified by a detector coupled to the HPLC. The most widely used detectors for HPLC analyses are UV-Vis, FL, diode-array (DAD) and MS/MS [5,20,27,28,29]. Employing some of these detectors requires a pre- or postcolumn derivatisation process because amines lack a suitable chromophore or fluorophore group. The most widespread prederivatisation agent is DNS-Cl [30,31], which is suitable for FL and UV-Vis detection, and has the advantage of generating stable derivatised compounds and good recovery with high sensitivity and resolution without interference [6]. Other precolumn derivatisation agents used in HIS analysis in food products are benzoyl chloride [32], dabsyl chloride (DBS-Cl) [33], 9-fluorenylmethoxycarbonyl chloride (FMOC-Cl) [34], naphthalene-2,3-dicarboxaldehyde (NDA) [33], coumarin-3-carboxylic acid [23], Py-Tag [35], 3,5-dinitrobenzoyl chloride [36], 2-napthyloxycarbonyl, trifluoromethyl, 6-aminoquinoyl-N-hydroxysuccinimidyl, diethylethoxymethylenemalonate and pyrene sulphonyl chloride [37]. A purification stage with n-hexane [20,28] or diethyl ether [30] has also been applied. For HIS postcolumn derivatsation, o-phtalaldehyde (OPA) is usually employed, but it creates fewer stable derivatives than DNS-Cl or DBS-Cl because it reacts only with primary amines. The advantage of OPA derivatisation is that it can be used for post-column derivatisation at room temperature, without the need for any previous clean-up or separation steps [6,30]. The use of MS/MS detectors with HPLC can eliminate the derivatisation step, but derivatisation is often still preferred because it improves the resolutions of polar amines in reversed-phase columns [12]. One of the main disadvantages of derivatisation is the use of organic solvents. To overcome this drawback, Gama & Rocha [38] have developed a sequential injection chromatography method with UV detection for the determination of underivatised BAs, including HIS, with a LOD of 0.500 mg/L. This method requires the use of untraditional columns like silica-based cyanopropyl and pentafluorophenyl columns.

The most frequently reported separation was based on reversed-phase chromatography including C18-type (octadecylsilane) columns because they allow adequate analyte retention and separation [39,40]. However, some authors refer to C8 (octylsilane) columns in their methods [29]. C8 columns have shorter alkyl chains and are less hydrophobic than C18 columns, which offer better retention for certain polar compounds like HIS without having to resort to a more specialised column. C18 and C8 columns usually have a stationary phase with a particle size of about 3–5 μm. However, particle sizes smaller than 2 μm have been reported for Ultra-Performance Liquid Chromatography (UPLC) [41]. UPLC columns significantly improve method performance, especially in terms of resolution, efficiency, separation capacity and speed, compared to conventional HPLC [15,42]. In practical terms, conventional HPLC methods employing 4–5 μm particles typically present elution times ranging from 20 to 60 min under back pressures below 200 bar, whereas UPLC methods using sub-2 μm particles (1.7–1.8 μm) operate at higher pressures (700–800 bar) and achieve separation in less than 10 min, while also significantly reducing solvent consumption [43]. In fact, reductions of up to 59% in both elution time and eluent consumption have been reported when switching from conventional HPLC to UPLC under comparable conditions.

Finally, more selective chromatographic strategies have been proposed to overcome the limitations of conventional reversed-phase HPLC for highly polar compounds such as HIS. Hydrophilic Interaction Chromatography (HILIC), which employs polar stationary phases to enhance the retention of hydrophilic analytes under high-organic mobile phase conditions, has proven to be a suitable alternative for HIS determination, providing improved retention and resolution without the need for ion-pair reagents [1,9,20,28]. Lioupi et al. [44] demonstrated that HILIC enables the direct analysis of HIS in complex food matrices with high sensitivity and chromatographic efficiency, notably eliminating derivatisation and additional clean-up steps, thereby simplifying sample preparation and reducing reagent and solvent consumption. In the same context, mixed-mode chromatographic approaches have also emerged as powerful alternatives, as illustrated by Zhao et al. [45], who employed a trimodal stationary phase combining cation-exchange, reversed-phase and HILIC interaction mechanisms to achieve the direct determination of underivatized HIS in fish products, without ion-pair reagents or extensive purification, further reducing solvent use and analytical complexity.

Of all the articles included in this section, 50% use a UV-Vis detector in their analysis protocol, followed by MS/MS (27%) and FL (23%) detectors. Some authors compare the performance of detectors coupled to HPLC. Sentellas et al. [42] concluded that HPLC methods with UV and fluorescence detection are fully compatible with HIS levels in food samples; however, MS detection has become very popular due to its excellent performance and the unambiguous identification of analytes. Nevertheless, Francisco et al. [16] reported that the UV detector shows lower LODs and limits of quantification (LOQ) (0.170 and 0.500 mg/L, respectively) than for the FL detector (1.600 and 4.900 mg/L, respectively), which also improved the precision and recovery rate for HIS in fish and meat samples. Other publications, such as Rivoira et al. [33], compared the effectiveness of different derivatising agents in combination with distinct types of detectors. These authors examined the performance of HIS derivatisation with NDA and DBS-Cl in HPLC coupled to FL and thermal lens spectrometry (TLS) detectors. They stated that the lowest LOD and LOQ for HIS were obtained by HPLC-DBS-TLS, with respective values of 0.011 and 0.040 mg/L compared to HPLC-NDA-FL (values of 0.027 and 0.081 mg/L, for LOD and LOQ respectively). A very low LOD is relevant because histamine can be useful not only for confirming obvious contamination, but also for detecting early spoilage or mishandling before concentrations approach regulatory limits. It also improves reliability in complex seafood matrices, where low analyte levels and matrix interference can affect HIS quantification.

Ionic chromatography (IC) is also considered an effective method for the determination of inorganic ions. However, there are few studies on BAs analysis because the strong hydrophobic interactions between the protonated amines and stationary phases typically used in this method result in long retention times and low resolution for chromatographic peaks. Over the years, IC in combination with different detection systems have been tested for HIS analysis with comparable results to other more commonly used techniques [46]. Jastrzębska et al. [26] proposed a method for the BAs analysis in food samples based on isocratic elution on a cation-exchange column with conductometric detection (CD), with a LOD of 0.071 mg/L and recovery of 97%. Despite these results, this method was unsuitable for BAs determination in complex food matrices given the strong interactions between the protonated amino groups and the stationary phase of the column. Later, Kočar et al. [47] developed a method for determining underivatised BAs using IC-MS/MS. In this case, a LOD of 2 × 10^−5^ mg/kg was achieved for HIS with very good repeatability (5–10% RSD) and in many complex food samples. Tsiasioti and Tzanavaras [18] devised a HIS determination method based on postcolumn derivatisation for exchange chromatography coupled to a spectrofluorimetric detector with a LOD of 0.0189 mg/L, and good precision (1.6% RSD) and repeatability in the analysed fish samples.

Considering the studies analysed in this review, reversed-phase HPLC remains the most widely used technique for HIS determination in food samples due to its robustness, versatility and widespread availability. In recent years, UPLC has emerged as an evolution of conventional HPLC, offering clear advantages in terms of shorter analysis times, higher resolution and reduced solvent consumption, although its implementation is still less common. In contrast, IC has been less frequently applied for BA analysis, particularly in complex food matrices, mainly due to strong interactions between protonated amines and the stationary phase, which lead to long retention times and poor peak resolution. Despite some improvements, these limitations restrict its applicability compared to HPLC-based methods. Therefore, the selection of the analytical technique is not only related to performance, but also to practical aspects such as instrument availability, cost, and the specific requirements of the analysis, including the need for derivatisation or simplified sample preparation.

Despite the accuracy, sensitivity and reliability of chromatography, which makes it one of the most widely used techniques for HIS determination, it has several disadvantages. These techniques are often time-consuming due to the number of required steps before the chromatographic analysis (extraction, cleanup, derivatisation and concentration), require skilled personnel and the use of high-quality organic solvents, which increases the technique’s cost [6]. These disadvantages must be considered, and this is the main reason why other methodologies have been developed to overcome these drawbacks. Nevertheless, validated HPLC methods are still considered the best option for quality control of fish and fishery products and for demonstrating compliance with applicable legislation. These methods conform to current regulations, especially ISO 19343:2017, AOAC 977.13, or equivalent internal HPLC methods validated according to international criteria. However, the sample preparation and derivatization steps required in these procedures slow down analytical workflows, hindering their practical application in routine industrial quality control. In such applications, other methods based on the use of first-line screening tests and rapid tests would be preferable to HPLC techniques.

**Table 2:** Summary of the conditions of the different HPLC methods for histamine determination and performance parameters.

Analytical Technique	Matrix	Sample Treatment			Precision (%RSD)	Range (Method)	Linearity (R^2^)	Recovery (%)	LOD	LOQ	Ref.
		Derivatisation	Clean-Up/Purification	Range (Sample)							
HPLC-UV	Red wine and fish	DNS-Cl	dLLME	-	Intraday: 50 (3.1)–200 (3.8) mg/kg Interday: 50 (3.7)–200 (3.1) mg/kg	0.010–1 mg/kg	0.995	Intraday: 50 mg/kg: 93.3–200 mg/kg: 96.1 Interday: 50 mg/kg: 94.5–200 mg/kg: 98.9	0.0028 mg/kg	0.0096 mg/kg	[24]
HPLC-UV	Chinese mackerel fresh and canned products	DNS-Cl	Magnetic SPE (CoFe_2_O_4_/MEG)	16.1–56.1 mg/kg	2.76	1–50 mg/L	0.9999	99.1	0.0036 mg/kg	0.012 mg/kg	[25]
HPLC-UV	Chub mackerel fillets	Derivatisation was not required	-	25.3–135 mg/kg	Intraday: 1.49–3.49 Interday: 1.63	0.15–12 mg/L	0.9998	42.5 mg/kg: 91.8; 82.5 mg/kg: 87.5 65 mg/kg: 8.43	1.300 mg/kg	4 mg/kg	[45]
HPLC-UV	Fish sauce	DNS-Cl	-	18.8–244 mg/kg	Intraday:1 (9.1)–5.2 (4.7)–26 (0.7) mg/100 g	0.12–120 mg/L	0.9997	10 mg/kg: 85.8; 52 mg/kg: 88.5; 260 mg/kg: 87.6	4 mg/kg	14 mg/kg	[29]
HPLC-UV	Raw and canned tuna	DNS-Cl	-	0.86–1279 mg/kg	-	5–1500 mg/L	0.9936	-	0.003 mg/kg	0.010 mg/kg	[48]
HPLC-UV	Milkfish and Indian whiting	DNS-Cl	-	172.8–286.9 mg/kg	-	172.8–286.9 mg/	0.903; 0.91	-			[49]
HPLC-UV	Canned fish	DNS-Cl	Carbon Spheres QuEChERS	12–282.5 mg/kg	Intraday: 2.3	1–50 mg/L	0.9999	97.7%	0.0072 mg/kg	0.0977 mg/kg	[21]
HPLC-UV	Fish flesh	DNS-Cl	Toluene	24.01–200.30 mg/kg	Intraday: 25 (18.2)–100 (6.11)–220 mg/kg (3.20) Interday: 25 (32.23)–100 (93.10)–220 (91.05) mg/kg	25–100–220 mg/kg	-	25 mg/kg: 96.03; 100 mg/kg: 93.10; 220 mg/kg: 91.05	-	-	[50]
HPLC-UV	Canned fish, sausages, beef, and meat-based and tuna-based pet food	DNS-Cl	Salting-out-assisted liquid–liquid extraction	2.61–36.7 mg/kg	Intraday: (2.3); Interday: (9.4)	1–10 mg/L	0.998;	93.6%	0.17 mg/kg	0.5 mg/kg	[16]
HPLC-UV	Fish	Benzoyl chloride	-	0.22–168.36 mg/kg	Intraday: 3.2; Interday:4.4	0.5–100 mg/L	0.9991	Intraday: 5 mg/L: 94.5; 20 mg/L: 95.28	0.04 μg/kg	0.16 μg/kg	[32]
HPLC-UV	Tuna, sardine, kilka and mackerel	-	-	9.66–71.66 mg/kg	Intraday: 1.3	5–50 mg/L	0.9909	82	1.5 mg/kg	5 mg/kg	[40]
HPLC-UV	Cheese	Derivatisation was not required			1.49	1.4–100 mg/L	0.999	-	0.5 mg/kg	1.4 mg/kg	[28]
Indirect RP-HPLC-UV	Fish	-	-	-	Intraday: 1 (2)–4 (1.7)–3.62 (3.62)–16 (0.83) mg/kg	1–32 mg/L	0.9995	1 mg/kg: 86; 4 mg/kg: 87.25; 8 mg/kg: 96.62; 16 mg/kg: 86.62	1 mg/kg	3 mg/kg	[17]
Narrow-bore HPLC-UV	Soya sauces, fish sauces, and dark malt beverages	Coumarin-3-carboxylic acid	n -hexane	1–368.8 mg/kg	Intraday: 1.1125 (3.8)–16.6875 (3) 38.9375 (5.3) mg/L Interday: 1.1125 (5.2)–16.6875 (2.5)–38.9375 (1.6) mg/L	0.333–44.46 mg/L	0.992	1.1125 mg/L: 2.8; 16.6875 mg/L: 2.1; 38.9375 mg/L: 2.2	0.111 mg/L	0.333 mg/L	[23]
HPLC-UV	Vinegar, baijiu, and chub mackerel	-	-	103–487 mg/kg	Intraday: 2 (4.9)–5 (4) mg/L	0.1–10 mg/L	0.9999	2 mg/L: 98.2; 5 mg/L: 99.1	10 mg/kg	33 mg/kg	[51]
HPLC-UV-DAD	Fish products	DNS-Cl	-	<3–>720 mg/kg	Intraday: 122 (1.6)–233 (2.1)–375 (2.1) mg/kg Intermediate precision: (2.7)	0–720 mg/L	0.9998	122 mg/kg: 100; 233 mg/kg: 97; 375 mg/kg: 103	3 mg/kg	10 mg/kg	[14]
HPLC-DAD	Anchovies	DNS-Cl		0.27–51.0 mg/kg	-	-	0.9980	-	0.06 mg/kg	0.20 mg/kg	[52]
HPLC-FL	Canned fish, sausages, beef, and meat-based and tuna-based pet food	DNS-Cl	Salting-out-assisted liquid–liquid extraction	2.61–36.7 mg/kg	Intraday: (4); Inter-day: (10)	1–10 mg/L	0.9993	111	1.6 mg/kg	4.9 mg/kg	[16]
HPLC-FL	Canned mackerel	OPA	SPE	UV: 10.59–23.44 mg/kg; FL: 6.67–25.10 mg/kg	Intraday: 12.5 (1.41)–25 (2.24)–50 (1.63)–100 (1.37)–200 (1.38) mg/kg	12.5–200 mg/L	0.999981	12.5 mg/kg: 98.49; 25 mg/kg: 98.60; 50 mg/kg: 98.72; 100 mg/kg: 99.02; 200 mg/kg: 98.88	1.8 mg/kg	UV: 8 mg/kg; FL: 5 mg/kg	[27]
HPLC-FL	Fresh tuna	OPA	-	2.06–83.73 mg/kg	Intraday: 10 (2.3); 20 (2.6); 30 (2.3) mg/L Interday: 10 (6.1); 20 (6.2); 30 (5) mg/L	0.2–50 mg/L	0.9996	80–110	0.03 mg/kg	0.09 mg/kg	[53]
HPLC-FL	Fish products	FMOC-Cl	-	0.00003–0.00116 mg/kg	Intraday: (1.73); Interday: (7.38)	0.16–5 mg/L	0.9998	103	0.10 mg/L	0.30 mg/L	[34]
Column-Switching HPLC-FL	Fish meat and fermented foods	DNS-Cl	Diethyl ether	2.89–22,368.18 mg/kg	-	0.0901–90.02 mg/L	0.9996	17.38 mg/L: 68.54; 34.73 mg/L: 83.78 69.47 mg/L: 83.04	0.0185 mg/L	0.0617 mg/L	[30]
UPLC-FL	Fermented food and fresh fish	OPA	-	0.9–164.4 mg/kg	Intraday: 100 (4.5) mg/kg	0.1–10 mg/L	Between methods (cITP-CZE & UPLC): 0.996	-	0.30 mg/kg	1 mg/kg	[15]
UHPLC-FL	Yellowfin tuna	OPA	-	9800–54,114.8 mg/kg	Intraday: (6.5); Interday: (6.94)	10–60 mg/L	0.9999	10 mg/L: 97.52; 30 mg/L: 89.68; 60 mg/L: 89.75	2.4 mg/kg	8 mg/kg	[54]
UHPLC-FL	Tuna	DNS-Cl	-	150–625 mg/kg	Intraday: 25 (11.6); 250 (5.4); 750 (9.1) mg/kg Interday: 25 (14.2); 250 (7.9); 750 (8.8) mg/kg	25–750 mg/L	0.9887	25 mg/kg: 80; 250 mg/kg: 95; 750 mg/kg: 103	2.71 mg/kg	8.31 mg/kg	[41]
HPLC-NDA-FL	Wine	OPA, NDA, DBS-Cl	-	0.08–0.09 mg/kg	-	LOQs–1.2 mg/L	0.985–0.997	93–112	0.027 mg/kg	0.081 mg/kg	[33]
HPLC-DAD	Cheese, fish and fishery products, and meat and meat products	DNS-Cl	-	0.1–98.8 mg/kg	Intraday: (2.6)	2.5–500 mg/L	1	Intraday: 100/250/500 mg/kg: 99	0.1 mg/kg	0.3 mg/kg	[5]
HPLC-DAD	Fish	DNS-Cl	-	0.36–52.62 mg/kg	-	0.05–20 mg/L	0.9999	-	0.30 mg/L	0.91 mg/L	[39]
HPLC-MS/MS	Fish and fish products	Py-Tag	-	2–367 mg/kg	Intraday: LOQ (3.22); 50 (2.8)–100 (2.34) mg/kg	0.0001–0.040 mg/L	>0.999	LOQ: 94.2; 50 mg/kg: 96.2 100 mg/kg: 962%	0.61 mg/kg	2 mg/kg	[35]
HPLC-MS/MS	Salted mackerel fillet	-	Ion-pair SPE	<LOQ/nd	Intraday: 2 (2.1)–7.5 (1.5) mg/kg Inter-ay: 2 (3.3)–7.5 (3.7) mg/kg	0.1–12.5 mg/L	1	2.5 mg/kg: 117.6; 5 mg/kg: 114.9	0.01 mg/kg	0.05 mg/kg	[22]
HPLC-MS/MS	Whey powder	-	-	0.089–0.413 mg/kg	Intraday: 5 (4.4)–10 (3.9)–25 (3.8) ng/mL Interday: 5 (6.7)–10 (5.9)–25 (5.8) ng/mL	0.0016–1 mg/L	0.9969	64	0.47 mg/kg	1.6 mg/kg	[42]
HPLC-MS/MS	Aquatic products	-	n-hexane	“Less than the USA and EU acceptable levels”	Intraday: (2.42) Interday: (6.17)	0.020–2 mg/L	0.9916	2.5 mg/kg: 76.2–111.3 5 mg/kg: 70.9–105.7 12.5 mg/kg: 75.5–102.3	1 mg/kg	2.5 mg/kg	[20]
HPLC-MS/MS	Seafood, liquor, fermented meat, vegetable and dairy products	-	n-hexane	0.22–33.14 mg/kg	Intraday: (3.66) Interday: (9.74)	0.001–0.1 mg/L	0.9995	0.25 mg/kg: 78.7–114.3 0.5 mg/kg: 70.9–104.5 1.25 mg/kg: 75.5–111.2	0.1 mg/kg	0.25 mg/kg	[28]
HPLC-MS/MS	Canned and fresh tuna	Derivatisation was not required	-	0.45–2.85 mg/kg	Intraday: 1 (2.0)–50 (1.1)–100 (3.3) mg/kg Interday: 1 (1.0)–50 (1.2)–100 (3.5) mg/kg	0.0625–150 mg/kg	0.9948	1 mg/kg: 93.5 50 mg/kg: 113 100 mg/kg: 103	0.019 mg/kg	0.0625 mg/kg	[55]
UPLC-MS/MS	Fish	-	-	2.3–15.4 mg/kg	Intraday: (0.12) Interday: (1.17)	0.4–3 mg/L	0.9979	50 mg/kg: 82.88; 75 mg/kg: 92.96; 100 mg/kg: 101.19	64 mg/kg	500 mg/kg	[19]
UPLC-MS/MS	Yellowfin tuna	-	-	0.3–332.24 mg/kg	Intra-day: (0.7–3); Inter-day: (3–4)	5–400 mg/L	>0.9995	98–103	0.30 mg/kg	1 mg/kg	[9]
IC-MS/MS	Fresh fish and processed fish products	-	-	0.3–226.9 mg/kg	Intraday: 500 (10) ppb; 5000 (5) ppb	0.03–10 mg/L	0.9982	100	0.00002 mg/kg	0.00006 mg/kg	[47]
IC-CD	Fresh and fruit juices, meat products, and smoked fish	-	Carrez solutions	1.45–335 mg/kg	Intraday: (0.56–1.97) Interday: (3.6)	0.5–5; 5–100 mg/L	0.9991; 0.9994	98	0.071 mg/kg	1.63 mg/kg	[26]
HPLC-DBS-TLS	Wine	OPA, DBS-Cl	-	0.08–0.09 mg/kg	-	LOQ–1.2 mg/L	0.985–0.997	93–112	DBS-TLS: 0.011; DBS-DAD: 0.085 (mg/kg)	DBS-TL: 0.040; DBS-DAD: 0.282 (mg/kg)	[33]
Cation exchange chroma-UV	Canned and frozen fish and sauces	OPA	-	<LOQ–1370 mg/kg	Intraday: (0.2); Interday: (4.6)	0.0556–1.667 mg/L	0.994	91.3–117.9	0.0189 mg/kg	0.0556 mg/kg	[18]

#### 3.2.2. Non-HPLC Methods

Table 3 summarises the conditions of the different chromatographic methods (except for HPLC) applied to HIS determination, as well as their performance parameters. Of these chromatographic methods, GC was one of the most employed. As a chromatographic method, it has the aforementioned disadvantages; however, organic solvent consumption is considerably reduced. Huang et al. [56] employed SPME coupled with GC and MS for BAs determination in fish samples. They used isooctane as extraction solvent and isobutyl chloroformate (IBCF) as derivatization reagent and compared performance of this protocol to dLLME, HS-SPME and Ion-pair extraction, concluding that LODs of BAs by this method were acceptable, and the extraction time was shorter than other extraction procedures. Munir et al. [57] analysed fish samples by gas chromatography coupled with flame ionisation detection (GC-FID) and derivatisation employing N,O–bis (trimethylsilyl) acetamide and trimethylchlorosilane (BSA+TMCS). The LOD of this method for HIS was 1.460 μg/L.

Thin-layer chromatography (TLC) is also employed for HIS analysis. This method can be used as either a semiquantitative method or a basic screening qualitative method due to its analysis speed, low cost and simplicity [48], and also for HIS quantification, as reported by several authors [58,59]. Kounnoun et al. [58] developed and validated a densitometric-TLC method for HIS determination in fish samples using DNS-Cl derivatisation, obtaining a LOD 4.400 mg/kg. Lu et al. [59] developed a rapid and ultrasensitive portable technology by combining azo-derivatised TLC with Surface-Enhanced Raman Spectroscopy (SERS). The proposed method yielded a LOD of 2.8 × 10^−5^ mg/L with the signal-to noise ratio at 3. Some TLC protocols include a purification step with toluene [58], and derivatisation with Pauly’s reagent, which consists of two reagents: A-1 (20 mM sulfanilic acid in HCl 1 M solution) and A-2 (200 mM sodium nitrite solution) [60]. TLC is not an expensive method and the results can be quickly obtained, but this technique is easily affected by the presence of other BAs with loss in accuracy, linearity, and applicability [30]. Recent developments have significantly expanded the analytical capabilities of TLC-based methods. Zhang et al. [61] reported a novel laser ablation dielectric barrier discharge TLC-MS (LA-DBD-TLC-MS) approach that enables the quantitative determination of BAs in fishery products with analytical performance comparable to HPLC-MS/MS. By integrating TLC separation with automated laser ablation, ambient plasma ionisation and MS detection, this methodology overcomes the traditional limitations of TLC, minimising manual handling and analytical variability while achieving high sensitivity and robustness. Furthermore, compared to conventional methods which require derivatisation, this system provides a simpler, higher-throughput and more cost-effective solution for BA analysis. These advances demonstrate that modern TLC-based platforms can evolve from screening tools to reliable quantitative techniques for HIS analysis in complex food matrices.

**Table 3:** Summary of the conditions of different chromatographic (non-HPLC) methods applied to histamine determination and performance parameters.

Analytical Technique	Matrix	Sample Preparation			Precision (%RSD)	Range (Method)	Linearity (R^2^)	Recovery (%)	Chromatographic conditions	LOD	LOQ	Ref.
		Derivatisation	Clean-Up/Purification	Range (Sample)								
GC-MS/MS	Mackerel and cured fish	IBCF	SPME	-	Intraday: 3.7–7.2; Interday: 11.2–14.3	-	0.9647–0.9991	78.9–110	Column/Stationary phase: RXi-5MS column (30 m × 0.25 mm). Carrier: Helium at 1.33 mL/min. Injection: 270 °C; splitless mode. Detection: MS/MS.	0.00298–0.0453 mg/kg	0.00985–0.14950 mg/kg	[56]
GC-FID	Fish and fish products	BSA+TMCS	-	0.37–5.96 mg/kg	Intraday: 0.77; Interday: 0.78	25–150 mg/L	0.9999	105.71	Column/Stationary phase: HP-5 phenylmethylsiloxane (30 m × 0.25 mm, 0.25 µm). Carrier: Hydrogen. Detection: FID and MS.	1.46 mg/kg	4.38 mg/kg	[57]
TLC-Densitometry Method	Fish and fish products	DNS-Cl	Toluene	11.9–39.23 mg/kg	Intraday: 6.25 (4.82); 12.5 (4.09); 25 (3.78); 50 (3.90); 100 (2.19) mg/kg Interday: 6.25 (11.16); 12.5 (5.37); 25 (6.35); 50 (7.29); 100 (5.80) mg/kg	3.75–50 mg/L	0.9995	6.25 mg/kg: 93.18 12.5 mg/kg: 105.28 25 mg/kg: 104.21 50 mg/kg: 97.33 100 mg/kg: 103.70	Column/Stationary phase: TLC aluminium sheets with silica gel 60 F254. Mobile phase: chloroform-triethylamine (6:4, *v*/*v*). Detection: UV at 365 nm.	4.4 mg/kg	13.2 mg/kg	[58]
TLC	Fish	-	-	47.7–77.2 mg/kg	-	0.05–1 mg/L	0.835	-	Column/Stationary phase: Warm plates sprayed with 3% (*v*/*v*) ninhydrin in methanol. Purple indicates the presence of BAs. Mobile phase: acetone and ammonium hydroxide (95:5 *v*/*v*).	-	-	[48]
TLC-MS	River perch, turbot, genuine porgy and salmon	Derivatisation was not required	-	164.86–463.34 mg/kg	Intraday: 100 mg/L (7.10–9.65) Interday: 100 mg/L (4.30)	1–1000 mg/L	0.9956	50 mg/L: 101.34 150 mg/L: 94.12 500 mg/L: 102.87	Stationary phase: Silica gel TLC plates (commercial precoated plates). Mobile phase: ethanol and ammonium hydroxide in a volumetric ratio of 3:1 Detector: MS: Direct laser ablation of separated spots from the TLC plate, followed by dielectric barrier discharge ambient ionisation	0.28 mg/L	0.924 mg/L	[61]
TLC-image analysis	Mackerel	-	-	31.27–4212.53 mg/kg	Intraday: 200 (2.76); 400 (1.55); 800 (1.07) mg/L	10–1 20 mg/L	0.9987	200 mg/L: 93.78 400 mg/L: 96.32 800 mg/L: 98.24	Column/Stationary phase: TLC plate precoated with silica gel G (10 × 20 cm, 0.25 mm layer). Conditions: Spray TLC plates with NaOH/ethanol, develop, and spray with diazonium reagent. Detection: TLC plate is photographed and coloured spots are digitised into optical density.	14.03 mg/kg	42.09 mg/kg	[62]
HPTLC modified with self-visualisation nanomaterials	Saury	-	-	85.7 mg/kg	-	1.25–160 µg/mL	0.9805	-	Column/Stationary phase: TLC strips. Conditions: TLC strips inside the migration chamber and washed with methanol/water (*v*/*v* = 95:5). Detection: TLC sampler to print dispersed ninhydrin@TiO_2_ composite (10.0 mg/mL).	5 mg/kg	16.5 mg/kg	[63]
TLC-SERS	Tuna	Pauly’s reagent	-	-	-	10–500 mg/L	0.968	-	Column/Stationary phase: Diatomaceous earth TLC plate Process: The TLC plate is developed with mobile phase eluent for 10 min and dried in an oven for 1 min. Detection: Pauly’s reagent for visualisation. Au-NPs solution on the spot by drop casting. A portable Raman spectrometer (excitation laser at 785 nm) obtains SERS signals.	10 mg/kg	30 mg/kg	[60]
Portable TLC-SERS	Tuna	Pauly’s reagent	-	0.05–1258 mg/kg	-	0.1–100 mg/L	0.9757	106–112	Column/Stationary phase: Diatomite TLC Process: Eluent is a mixture of ethanol and ammonium hydroxide (3:1, *v*/*v*). TLC plates are sprayed with Pauly A reagent and then with Pauly’s B reagent. Before SERS measurements, 2 μL of Au colloid are cast to spots of analytes on TLC plates. Detection: A red spot corresponds to HIS.	2.8 × 10^−5^ mg/kg	9.2 × 10^−5^ mg/kg	[59]

### 3.3. Spectroscopic Methods

Table 4 summarises the spectroscopic methods applied to HIS determination, along with their analysis conditions and performance parameters. Spectroscopy measures the interaction between radiation and molecular structure, specifically the imidazole ring and the ethylamine side chain. Depending on the nature of the interaction, there are several methods: absorption, fluorescence, phosphorescence, scattering, emission and chemiluminescence spectroscopy.

Altunay et al. [64] use Flame Atomic Absorption Spectroscopy (FAAS) for HIS analysis in fish samples, dairy products and alcoholic beverages. They combined ultrasonic-assisted cloud point extraction (UA-CPE) for the HIS preconcentration from the sample and indirect HIS determination by FAAS. The proposed method was adequate in terms of sensitivity (LOD of 2.5 × 10^−5^ mg/L) and precision (RSD lower than 4.8%), and had a high sensitivity enhancement factor of 143. These authors also tested the proposed method in real samples (anchovies, canned tuna, canned sardines and canned mackerels) obtaining good recovery rates ranging from 97 to 103%. According to these authors, this methodology is advantageous in terms of low LODs, good precision and reduced use of toxic organic solvents compared to other analytical methods (Table 3).

Near-Infrared Spectroscopy (NIR) is also applied in the food industry as a qualitative method to analyse food quality and detect food fraud in different food products. For fish and fishery products, this technique has also been applied for freshness evaluation [65]. However, analysing compounds at very low concentrations using NIR is difficult due to the low sensitivity of the method and the interference caused by the water present in the sample, which might interfere with the results [47]. Mobarez et al. [66] developed an NIR dye reactive to BAs for HIS measurement, with a LOD of 0.010 mg/kg using standard solutions. However, when it was applied to real samples (shrimp samples), this method was useful for determining the total content of BAs, but failed to measure HIS. Also, Ghidini et al. [47] evaluated the feasibility of NIR to directly quantify HIS content in raw and processed tuna, concluding that their methodology was able to detect samples whose HIS content may be high. However, when they compare NIR and HPLC performances, NIR method showed higher LOQ and LOQ values than HPLC. According to these results, NIR technique could provide rapid and non-destructive analysis of many samples, increasing the chances of detecting non-compliant ones that can later be confirmed with reference methods, being an excellent tool for short-term screening and semi-quantitative analysis. In this sense, the NIR technique could provide quick and reliable data, playing a key role in ensuring food safety and avoiding food losses.

Supporting techniques like vibrational spectroscopy have been developed to quantify HIS in fish. For example, SERS has proven potential high functionality by amplifying the HIS signal via plasmon resonance and preconcentration steps to overcome the low sensitivity associated with this technique [67]. Alizadeh et al. [68] developed a novel technique to determine HIS by ion mobility spectroscopy (IMS) combined with headspace solid-phase microextraction (HS-SPME-IMS), based on the separation and identification of ionised molecules when they drift in the gas phase depending on their mobility in an electric field at atmospheric pressure. A LOD of 3 × 10^−3^ mg/kg, good precision (5.7% RSD) and recovery (94–96%) were achieved with this methodology for canned fish samples in a total time of 42 min. More recently, vibrational spectroscopy coupled with chemometric and machine learning tools has been proposed as a rapid screening strategy for HIS determination. Sánchez-Parra et al. [69] demonstrated that Fourier Transform Mid-Infrared Reflectance Spectroscopy (FT-MIR) can be used for the direct, non-destructive quantification and classification of HIS levels in fresh tuna without prior sample treatment, applying partial least squares-discriminant analysis (PLS-DA), k-nearest neighbours (KNN), and support vector machine (SVM) algorithms to discriminate samples according to regulatory thresholds. Robust partial least squares regression (PLSR) models were developed for quantitative prediction, achieving excellent performance with R^2^ higher than 0.97 in prediction and up to 0.99 in cross-validation, together with low prediction errors (RMSEP as low as 5.840 mg/kg), highlighting the strong correlation between spectral data and HIS concentration. Similarly, Currò et al. [70] reported the successful application of NIR combined with advanced machine learning techniques for HIS screening in frozen-thawed tuna. Modified partial least squares (MPLS) regression showed good predictive performance for quantification (R^2^ CV = 0.88; R^2^ P = 0.74), while SVM classification models achieved high accuracy, reaching 100% in binary classification (presence/absence) and 93% in multiclass discrimination (>100 mg/kg). Although these approaches do not replace conventional confirmatory methods, such as HPLC, they provide rapid, non-destructive, and environmentally friendly alternatives for high-throughput industrial screening and targeted control.

Fluorescence spectroscopy (also called fluorimetry) is based on the emission of light by molecules previously excited by an external radiation source, typically ultraviolet light. Upon excitation, electrons reach a higher energy state and subsequently return to the ground state, emitting radiation that can be measured with high sensitivity. This approach is widely applied to HIS determination after derivatisation with fluorescent reagents. However, fluorescent derivatives of HIS, particularly those formed with OPA, are known to be unstable, with signal decay occurring within minutes after reaction. To overcome this limitation, Boukadida et al. [71] developed an improved fluorimetric method based on OPA derivatisation in the presence of non-ionic surfactants (C_12_E_23_) in water/ACN media. The solubilisation of the OPA–HIS complex within the polar region of surfactant micelles significantly stabilises the derivative and enhances fluorescence emission, improving both signal intensity and temporal stability, with a good LOD and LOQ (0.576 and 1.920 mg/L, respectively), as well as excellent linearity (R^2^ = 0.9978) and repeatability (CVr = 7.86%). Additionally, Geng et al. [72] proposed a simple fluorescein-based probe (FCHO) for rapid (<1 s) and selective HIS detection via an imine (Schiff base) formation reaction. The probe, synthesised in a single step by introducing an aldehyde group into fluorescein, exhibits quenched fluorescence in its closed lactone form. Upon reaction with HIS, ring opening occurs and fluorescence is significantly enhanced, enabling rapid quantitative detection. Coupled with a paper-based test chip, the system allows colour-resolved, highly selective recognition and point-of-care testing (POCT). Notably, the probe was successfully applied for visual imaging of HIS changes in fish samples and, for the first time, enabled direct in situ imaging of HIS distribution in whole spoiled fish.

To further improve the reliability of fluorescence-based screening, recent advances have focused on ratiometric dual-mode systems that combine fluorescence and colorimetric responses. In this context, Zhang et al. [61] developed a self-assembled dipeptide-based ratiometric fluorescent probe for the detection of BAs, including HIS. The system is based on the coordination-driven self-assembly of L-carnosine with Zn^2+^, subsequently coupled with methyl red to generate a dual-emission nanoprobe (400 and 610 nm). Upon interaction with HIS, the fluorescence intensity ratio (F400/F610) changes proportionally to analyte concentration, minimising environmental and instrumental interferences through internal self-calibration. The method showed good linearity within 1–50 mg/L and a low LOD (0.380 mg/L for HIS), together with good stability and anti-interference performance. Notably, the probe was integrated into paper-based test strips enabling visual colour-resolved detection via RGB analysis, demonstrating its potential as a rapid, sensitive and practical spectroscopic tool for on-site HIS screening.

Interestingly, monitoring fish freshness is progressively moving from laboratory-based instrumentation to mobile-screen platforms, enabling portable and user-friendly on-site assessment. In this context, Sun et al. [73] developed an innovative fluorescence-based system for HIS detection that combines a phenothiazine-derived “OFF–ON” probe (MPZ) with a dedicated smartphone application. The probe rapidly detects HIS (response time = 7 s; LOD = 0.311 mg/L) through a deprotonation mechanism, generating a distinct red fluorescence signal (λ_em = 620 nm) together with visible colour changes. To overcome the subjectivity associated with naked-eye interpretation, the authors designed the “Visual Evaluation” app, which captures images of the sensing tag, automatically extracts RGB values, performs data processing and fitting, and stores results, thereby transforming colour variation into quantitative analytical information. This integration of a fluorescent probe with a mobile application significantly enhances accuracy, reproducibility, and portability, representing a major advance toward reliable POCT HIS screening in fish products.

**Table 4:** Summary of the conditions of different spectroscopic methods applied to histamine determination and performance parameters.

Analytical Technique	Matrix	Sample Preparation		Precision (%RSD)	Range (Method)	Linearity (R^2^)	Recovery (%)	Detection Conditions/Sensing Mechanism	LOD	LOQ	Ref.
		Clean-Up/Purification	Range (Sample)								
FAAS	Fish, dairy products, and alcoholic beverages	UA-CPE	0.0078–0.055 µg/kg	Intraday: 0 (3)–0.005 (3.6)–0.020 (4.1) mg/L Interday: 0 (1.9)–0.005 (2.5)–0.020 (2.8) mg/L	0.0008–0.170 mg/L	0.992	0.005 mg/L: 97.3–102.6% 0.020 mg/L: 97.7–101.3%	Source: Iron hollow cathode lamp. Detection: 248.3 nm.	2.5 × 10^−5^ mg/kg	8.3 × 10^−5^ mg/kg	[64]
NIR	Shrimp	Carrez solution	838.1–2412.0 mg/kg	-	1.111–111.15 mg/L	>0.96	-	Source: home-built electrospinning. Detection: luminescence spectrometer at 600 nm (ex) and 4–8 nm (em).	10 mg/L	30.02 mg/L	[66]
NIR	Raw and processed Tuna		10–1694 mg/kg	Intraday: Raw 4.9; Processed 3.2 Interday: Raw 8.1; Processed 4.8	10–1000 mg/L	Raw tuna: 0.987; Processed tuna: 0.989	10 mg/kg: 86.2–89.4%; 50 mg/kg: 98.2–104.8%; 100 mg/kg: 98.4–102.6%	Detection: spectrum recorded at a 1 nm interval for 1000–2500 nm.	Raw: 4 mg/kg; Processed: 2 mg/kg	Raw: 12 mg/kg; Processed: 8 mg/kg	[47]
NIR	Frozen-thawed tuna		0–≥200 mg/kg	-	-	0.9800	-	NIR spectra acquired in reflectance mode (400–2500 nm, 0.5 nm intervals) using a NIRS™ DS2500 benchtop spectrometer; samples placed in standard circular ring cups.	-	-	[70]
DART-MS	Tuna and yellowtail fillets		1.6–40.7 mg/kg	-	0.5–50 mg/L	0.96	151.1–619.9%	Source: DART ion source with He as the ionising medium.	-	-	[74]
Spectrophotometry	Canned fish products	TC-IL-ME	62.8–390 mg/kg	Intraday: 0.010 (3.7)–0.100 (3.1)–0.300 (2.5) mg/L Interday: 0.010 (4.1)–0.100 (3.4)–0.300 (2.8) mg/L	0.002–0.400 mg/L	0.9979	0.10 mg/L: 95–96.1%; 0.100 mg/L: 96.4–7.3%; 0.300 mg/L: 97.2–97.7%	Detection: 368 nm.	6.5 × 10^−4^ mg/L	2 × 10^−3^ mg/L	[75]
UV-Vis spectrophotometry	Fish and meat products	Ultrasound-assisted low-density solvent-based dLLME	0.00634–0.5667 mg/kg	Intraday: 0.050 (4.5)–0.250 (3.5) mg/L Interday: 0.050 (5.6)–0.250 (5.1) mg/L	0.00575 mg/L	0.9945	0.2 mg/L: 95.2–102.8%	-	1.35 × 10^−3^ mg/L	4.52 × 10^−3^ mg/L	[76]
IMS	Canned fish samples	HS-SPME	0.036–0.064 mg/kg	Intraday: 5.7	0.030–0.300 mg/L	0.9955	0.050 mg/kg: 94%; 0.100 mg/kg: 96%	-	3 × 10^−3^ mg/kg	1.1 × 10^−2^ mg/kg	[68]
Fluorometry	Fresh yellowfin tuna, mahi-mahi, fresh Spanish mackerel	Ion-exchange column, eluted with deionised water. OPA derivatisation	23.3–103.2 mg/kg	25 (1–2)–50 (1–2)–100 (1–3) mg/kg	5–250 mg/L	0.9991	25 mg/L: 94–105%; 50 mg/L: 94–102%; 100 mg/L: 95–100%	Detection: 360 nm (ex) and 430 nm (em).	0.2–0.5 mg/kg	0.6–1.3 mg/kg	[77]
Fluorimetry	Fish and anchovies	Elution of sample filtrates	8.29–67.63 mg/kg	-	-	-	-	Detection: Fluorescence intensity recorded after 1.5 h at 450 nm.	-	-	[78]
Fluorimetry	Wine	-	0.51–3.70 mg/L	Repeatability: CV_r_ = 7.86%	0.7–13 mg/L	0.9978	75–99%	Cary Eclipse Fluorescence spectrofluorometer controlled by the Cary WinFLR software. Sample holders: quartz fluorescence cuvettes of 1 cm optical path length. Data mode: Fluorescence, Scan setup: Emission from 350 to 600 nm, Excitation wavelength: 340 nm, Excitation slit: 5 nm, Emission slit: 5 nm, PMT voltage: Medium).	0.576 mg/L	1.920 mg/L	[71]
Fluorimetry	Crucian carp, prawn, and pork	-	22.12–118.97 mg/L	Crucian carp: 22.23 mg/L: 1.88; 66.69 mg/L: 4.35; 116.71 mg/L: 0.5 Prawn: 22.23 mg/L: 2.77; 66.69 mg/L: 2.65; 116.71 mg/L: 0.97	0.111–4.446 mg/L	0.9909	Crucian carp: 22.23 mg/L: 87%; 66.69 mg/L: 96.4%; 116.71 mg/L: 101% Prawn: 22.23 mg/L: 95.3%; 66.69 mg/L: 103%; 116.71 mg/L: 98.8%	FCHO probe was designed and synthesised via one-step method. The specific Schiff base reaction between the aldehyde group on FCHO and the amino group on HIS and hydrogen bond formation between hydroxyl and imidazole could occur simultaneously.	5.67 × 10^−3^ mg/L	1.87 × 10^−2^ mg/L	[72]
Fluorimetry + Colorimetry	Fresh salmon		Visual detection	0–13.89 mg/L	0.9990	-	-	“OFF–ON” fluorescence response based on HIS-induced deprotonation of (MPZ), producing enhanced red fluorescence (620 nm) and measurable RGB colour changes via smartphone analysis	0.311 mg/L	1.027 mg/L	[73]
Fluorimetry + Ratiometric	Fish		Visual detection	1–50 mg/L	0.9809	-	-	Ratiometric fluorescence sensing based on dual-emission (400/610 nm) self-assembled dipeptide–methyl red nanoprobe; HIS induces charge transfer interactions that increase the F400/F610 ratio, enabling self-calibrated quantification	0.38 mg/L	1.25 mg/L	[61]
FT-MIR	Yellowfin Tuna		Screening method		0.9000	-	-	FT-MIR equipped with a Globar source and DLaTGS detector; spectra recorded in the 4000–600 cm^−1^ range at 4 cm^−1^ resolution, averaging 64 scans.	-	-	[69]

### 3.4. Enzymatic Methods

Table 5 summarises the different enzymatic methods applied to HIS determination. Of them, the most widely used is an ELISA test, based on the immunological antigen–antibody reaction [18]. Different ELISA kits have been developed, which were highly automated and allowed rapid analyses (<2 h) without using organic solvent (water extraction in fish samples). Examples of these tests were reported by Yang et al. [79], evaluating two different assessed test kits called “Histamine Food ELISA” and “HistaSureTM ELISA”. Both, based on the principle of competitive ELISA, were employed for HIS quantitative assays where derivatisation was not required. The HIS content in various fish products based on Food ELISA and HistaSureTM ELISA showed a good correlation between them (R^2^ = 0.9918). These methods were also well-correlated with the HPLC methodology (R^2^ = 0.9074 for Food ELISA and R^2^ = 0.9355 for HistaSureTM ELISA) [79].

Modifications in immunoassays were introduced to lower the LOD of HIS in seafood samples, such as sandwich ELISA proposed by Yang et al. [80], with a LOD of 5.86 × 10^−3^ mg/kg. HIS detection in fish samples based on indirect competitive ELISA (ic-ELISA) using an iron-cobalt co-doped carbon dots labelled HIS antibody, as proposed by Li et al. [81], resulted in a LOD of 0.50 mg/kg, which is still much lower than previously described commercial kits and HPLC methods.

Xu et al. [82] compared ELISA and Chemiluminescence Enzyme Immunoassay (CLEIA) to a conventional derivatisation HPLC-DAD analysis for HIS determination in food (seafood and wine) samples. Good correlation was found among both techniques (R^2^ = 0.987; R^2^ = 0.991, for ELISA and CLEIA, respectively). The difference in the HIS contents obtained in the three methods was not significant, and both immunoassays present LODs of 0.089 mg/L and 0.0734 mg/L, respectively, which reveals higher sensitivity than HPLC-DAD. These two developed immunoassays did not need a derivatisation step, reduced the use of organic solvents and reagents, shortened the analysis time and also had a lower LOD than most other immunoassays, as seen in Table 5.

Other enzymatic tests were developed and commercialised, such as the R-Biopharme AG RIDASCREEN^®^ Histamine kit, validated by the AOAC Research Institute in Meinhardt (2019). This test was based on HIS dehydrogenase that catalyses the oxidative deamidation of HIS in the presence of an electron carrier, which converts a dye into a colour product measured at 450 nm. This enzymatic test combined with the RIDA^®^ Sampel Decolorant kit (R-Biopharm AG, Darmstadt, Germany) reagents, used to eliminate colour pigments and interfering compounds, was available for accurate HIS quantification in fish products and other food samples (1.46–69.4 mg/kg) with a LOD and LOQ of 0.15 and 0.39 mg/L, respectively [83].

**Table 5:** Summary of the conditions of different enzymatic methods applied to histamine determination and performance parameters.

Analytical Technique	Matrix	Range (Sample)	Precision (%RSD)	Range (Method)	Linearity (R^2^)	Recovery (%)	Sensing Mechanism/Detection Conditions	LOD	LOQ	Ref.
Sandwich ELISA	Fish, prawn & crab	2.23–23.1 mg/L	(1.43 -11.7)	0.0117–1.50 mg/L	0.9942	80.19–108.3%	-	5.86 × 10^−3^ mg/L	1.934 × 10^−2^ mg/L	[80]
ELISA	Fish products	-	-	<1–7331 mg/kg	-	-	ELISA plates recorded at 490 nm.	0.30 mg/kg	1 mg/kg	[84]
ELISA	Fish products and soya sauce	8.4–112.5 mg/kg	10, 50, 100 mg/kg (0.4–18.5)	0.1–10 mg/L	0.987	10, 50, 100 mg/kg 74.2–131%	-	0.089 mg/L	0.2937 mg/L	[82]
ic-ELISA	Chilled and fermented fish	22.76–116.5 mg/kg	Intraday: 25 (0.17)–50 (0.28)–100 (0.11) mg/kg; Interday: 25 (0.12)–50 (0.33)–100 (0.2) mg/kg	2.5–150 mg/L	0.994	25 mg/kg: 91.07%; 50 mg/kg: 102.5%; 100 mg/kg: 116.5%	The mimic enzyme of Fe&Co co-doped CDs synthesised and labelled with HIS-Ab.	0.50 mg/kg	1.67 mg/kg	[81]
ELISA (FOOD ELISA, HistaSURE, Histagold)	Fish	1–320 mg/kg	**-**	5–200 mg/L	0.9074 & 0.9355	-	**-**	0.23 mg/kg	0.75 mg/kg	[79]
ELISA (HistaSure ELISA)	Fish	0.27–267.3 mg/kg	1.80–13.9	3–300 mg/L	0.9976–0.9996	86–99.8%	Substrate TMB/peroxidase reaction monitored at 450 nm.	0.44 mg/kg	1.31 mg/kg	[85]
ELISA (Neogen Veratox^®^)	Fresh/frozen, canned and ripened products	2.6–5542 mg/kg	<10%	2.5–250 mg/L	-	-	ELISA plates recorded at 620 nm.	0.76 mg/kg	2.5 mg/kg	[86]
Enzymatic (R-Biopharm AG RIDASCREEN^®^)	Fish, cheese & wine	1.46–69.4 mg/kg	1.5–11.1	1–20 mg/L	-	96–130%	ELISA plates recorded at 450 nm.	0.15 mg/L	0.39 mg/L	[83]
CLEIA	Fish products and soya sauce	8 -106.9 mg/kg	10, 50, 100 mg/kg (0.8–19.3)	0.1–10 mg/L	0.991	10, 50, 100 mg/kg 79.9–119%	-	0.0734 mg/L	0.2422 mg/L	[87]

### 3.5. Sensor Models

Traditional sensors are devices composed of four components: the target analyte, recognition element, signal transducer, and processor. Current advances in sensing technologies have enabled the development of novel applications across a wide range of fields, including the food sciences [88], providing a rapid, real-time and on-site detection of contaminants or quality indicators. Sensors can be classified by their transduction mechanism as optical and electrochemical [89], or by their recognition element, differentiating into biological (enzymes, peptides, proteins or antibodies), biomimetic (aptamers or nanozymes) and chemical-based recognition (metal and metal oxide nanoparticles, MIPs, Molecular Organic Frameworks (MOFs)). The most recent sensor developments applied to HIS determination, along with their performance parameters, are shown in Table 6.

Optical sensors can be further divided into fluorescent and colorimetric sensors, depending on the detection strategy applied. Recent advances in optical sensors for HIS analysis focus on improving specificity and enabling real-time monitoring through advanced nanotechnology and portable devices.

Fluorimetric sensors have emerged as highly sensitive tools for HIS detection of HIS. These sensors operate by measuring changes in fluorescence intensity or emission wavelength that occur when HIS interacts with a fluorescent probe or sensing platform. The incorporation of selective recognition elements, such as enzymes, aptamers, or MIPs, significantly enhances the selectivity toward HIS. Zhang et al. [90] developed a multiplexed fluorescence immunoassay, in which capture probes compete with HIS to combine corresponding signal probes. Compared to the traditional ELISA method, this sensor increases the sensitivity for HIS by about 280-fold with a LOD of 1 × 10^−5^ mg/L within a linear range of 1 × 10^−5^–0.100 mg/L. This new method used upconversion nanoparticles which, compared to the fluorescent materials in other sensors, possess interesting optical and chemical characteristics, such as low toxicity, higher stability, longer lifetime, better resistance to photobleaching, high stokes shift and non-autofluorescence. Other biosensors, like specific binding bioreceptors (peptides) and carbon dots developed by Shi et al. [91] offer significantly lower LODs than traditional methods (0.013 mg/L). In addition, carbon dots provide photostability, lower toxicity and good biocompatibility [92]. The fluorescent sensor created by Xu et al. [93], which is capable of generating a dual stimulus-response fluorescence based on dye covalently modified metal–organic frameworks, offers many advantages for HIS monitoring compared to other fluorescence sensors. This sensor also increases equipment stability by preventing transudation, as well as the detection mechanism (pH-induced energy transfer), which increases fluorescence within a wide pH range towards notorious colour changes. More recent advances in analytical sensing platforms highlight a strong trend toward the integration of nanostructured materials, selective recognition elements, and portable detection systems to improve sensitivity, selectivity, and practical applicability. One of the most notable trends across these studies is the extensive use of nanomaterials to improve sensor performance. Phoungsiri et al. [94] employed Alizarin complexone-modified gold nanoparticles for a dual-signal colorimetric and fluorometric sensor which was testing by using different types of fish products. This sensor enabled sensitive and selective detection of HIS, with a LOD 12.91 mg/L and 6.59 mg/L for colorimetric and fluorometric response, respectively, and a wide linear response range. Yang et al. [95] developed a copper nanoclusters in alginate-based hydrogel sensor for HIS detection. The proposed sensor shows a good linear correlation between fluorescence ratio and HIS concentration in the range of 0.33–066 mg/L with a detection limit of 1.85 mg/kg. Biomimetic recognition elements have gained interest in the latest sensors developments. Chen et al. [96] employed dsDNA-templated Cu nanoclusters (Cu-MOF, Ce-MOF, Mn-MOF) which was applied to determine HIS in sole and basa fish with satisfactory accuracy and precision and low LOD of 0.81 mg/L. Integrating fluorescent probes with MIPs enhances sensor selectivity, as artificial antibodies, providing lock-and-key recognition similar to natural receptors. Compared with biological receptors, MIPs offer high specificity, excellent thermo-chemical stability, and lower cost, making them increasingly important in the development of selective sensing platforms. Wang et al. [97] designed a novel molecularly imprinted ratiometric fluorescent sensor (OMQDs@BMQDs@MIPs) for the visible and fluorescent detection of HIS. This prototype was tested by using mackerel, packed codfish and canned tuna, obtaining good recovery dates (96–105%) and low LOD (2.4 × 10^−3^ mg/L). He et al. [98] employed MIPs as dispersive solid phase extraction sorbent to selectively extract HIS from canned tuna, followed by fluorescence detection using carbon quantum dots. The change in fluorescence was converted into specific RGB values using a portable UV light box combined with a smartphone.

A fluorometric and colorimetric method was developed by Wang et al. [99] for HIS detection in fish. The method was based on the fluorescence labelling of HIS-specific aptamer recognition via the quenching and optical properties of gold nanoparticles (AuNPs). The proximity of AuNPs and other magnetic nanoparticles causes a fluorescence resonance energy transfer (FRET) phenomenon that results in the quenching of the fluorescence signal in the detection system. The presence of HIS competes with AuNPs to capture the aptamer and to release it from the AuNP surface by inducing fluorescence recovery. With this technique, a LOD of 5.08 × 10^−4^ mg/L was achieved for HIS by using only water as the extraction solvent [99]. More recently, other nanomaterials have also been considered in colorimetric HIS sensor. Zou et al. [100] developed a HIS sensor using MOF-based nanozymes for the discrimination of BAs. This sensor achieved high sensitivity (LOD of 0.47 mg/kg) and selectivity for HIS detection, and also provided good results for monitoring fish freshness. A novel “off-on” colorimetric sensor for HIS analysis was developed by Wu et al. [101], based on Cu^2+^-mediated laccase-mimic activity of Mn_3_O_4_ nanoparticles. The LOD of the constructed sensor was as low as 0.148 mg/L and operated fast (20 min), suggesting it was suitable for on-site screening of HIS in food products.

Colorimetric HIS sensors are simple, low-cost, and well suited for rapid on-site screening, while fluorometric sensors provide superior sensitivity and analytical precision. Consequently, the choice between these approaches depends on the analytical requirements, with colorimetric sensors favoured for rapid field analysis and fluorimetric sensors preferred for highly sensitive laboratory-based measurements.

The development of new spectroscopic techniques has also rekindled interest in RAMAN spectroscopy as a tool to study surfaces and interfaces. One such phenomenon is SERS, in which a sample is irradiated with a powerful light source (usually a laser), and a small fraction of this radiation is scattered by certain molecules at wavelengths different from those of the incident radiation. Gao et al. [102] developed sensors based on the SERS technique using molecularly imprinted polymers and silver colloid SERS substrates, respectively. The extraction and purification protocols followed by the SERS analysis in both cases allow rapid, inexpensive HIS determination in fish samples.

Due to localised surface plasmon resonance (LSPR), gold and silver nanoparticles are very sensitive to changes in refractive or dielectric indices. Xu et al. [82] designed a multicolorimetric biosensor based on that etched between the hydrogen peroxide generated by the reaction of HIS and DAO and AuNRs, which causes a blueshift of the longitudinal peak of NRs. This reaction is accompanied by a colour change from green-blue to red and purple, where rapid visual HIS detection can be done with a LOD of 1.485 mg/L.

Electrochemical sensors can be classified depending on the detection strategy, and voltammetric, impedimetric, potentiometric, amperometric and piezoelectric sensors can be found for HIS detection. Thanks to the mechanical, physical, chemical and electronic properties that result from the coupling of electrochemical sensors and nanomaterials, the sensitivity and selectivity of techniques for the detection and quantification of very small amounts of HIS in food have improved [103]. It is possible to combine the nanomaterials making up the electrochemical sensor with an antibody or another receptor bioelement to form a biosensor because most of these NPs carry amine or carboxylic acid functional groups [89]. Wang et al. [104] report a molecularly imprinted electrochemiluminescence sensor based on Mxene (a new type of 2D transition metal carbide/carbonitride) and sodium ascorbate with a dual recognition and coordinated amplification carbon nitride nanosheets (CNNS) signal voltametric strategy. It increased HIS selectivity compared to other sensors because its LOD and LOQ was 1.9 × 10^−4^–5.7 × 10^−4^ mg/L, respectively, when using reference materials. However, in real samples, this sensor leads to lower recovery levels than chromatographic techniques. Salayani et al. [105] developed an electrochemical nanosensor based on two-dimensional Ti_3_C_2_ MXene/multi-wall carbon nanotube (MXene/MWCNT) signal amplification strategy and MIP technique. The proposed sensor showed a linear correlation between 1.0 and 280 mg/L, with a LOD of 0.83 mg/L.

Potentiometric sensors are designed based on the ability to detect variation in the electrical potential of an electrode when it comes into contact with HIS [106]. An example of this technique is found in the potentiometric biosensor developed by Basozabal et al. [107]. It consists of the incorporation of nanoMIPs into polyvinychloride membranes which was used to fabricate an ion-selective electrode with the ability to measure HIS in food in the presence of other biogenic amines.

Amperometric sensors detect the electric current through an electrode when an electric potential is applied. Pérez et al. [108] used DAO and HRP immobilised into PS/carbon nanotubes/ferrocene membranes to develop an amperometric bienzymatic biosensor for measuring HIS content. This sensor offers some advantages over traditional methods, such as a lower LOD (1.889 mg/L), a shorter analysis time, good reproducibility and repeatability, better sensitivity and a lower cost per analysis compared to reference methods like ELISA kits and chromatography.

Electrochemical impedance combines the analysis of the resistive and capacitive properties of materials. It is based on the perturbation of an equilibrium system by a small amplitude sinusoidal excitation voltage signal. All the substances within the electrochemical cell (i.e., resistors, capacitors and inductors) exhibit opposition to the movement of electrons and ions, which results in variation in impedance [109]. This technique is a sensitive indicator of a wide range of physico-chemical properties. An impedimetric biosensor was developed by Delle et al. [110], based on a short ultrathin graphene oxide line as a platform for detecting antigen–antibody binding events of HIS, with concentration ranges of 0.0111–0.111 mg/L through impedance changes within a low frequency range.

Piezoelectric sensors are a type of analytical technique that can measure molecular interactions on a surface by detecting changes in the resonance frequency of piezoelectric crystals due to mass changes. This method is divided into two types: quartz crystal microbalance (QCM) and surface acoustic wave (SAW) devices [111]. Dai et al. [112] employ a QCM sensor for measuring HIS based on molecularly imprinted Sol–Gel polymer, where an alternating voltage applied to crystal causes vibration and the resonance frequency is measured as it is introduced into an oscillation circuit. When a layer grows on the crystal surface, it alters oscillation frequency, which is directly proportional to the mass deposited on the crystal. This method has a LOD of 7.49 × 10^−4^ mg/kg with a regression coefficient of 0.9965 within a linear concentration range between 0.11 × 10^−2^ and 4.45 × 10^−2^ mg/L in spiked fish products [112].

Most recently developed sensors have succeeded in reducing LOD achieved using conventional techniques, reaching values as low as 2.48 × 10^−6^ mg/L for HIS detection in fish samples [113]. These systems can overcome the disadvantages of other methods, such as time consumption, derivatisation, skilled personnel required to operate in HPLC or the difficulty of producing affinity and specific antibodies in immunoassays [91]. Moreover, sensors have proven effectiveness in monitoring food samples in real time, and do not require any special skill to operate, and offer a fast response time, a wide linear range, long-term stability, reproducibility, selectivity and good accuracy [114,115].

Although sensor models are very promising for determining histamine in fish and fishery products, as they offer rapid analysis, high sensitivity, portability and the possibility of performing on-site tests, their current applicability in routine official control laboratories remains limited by various practical restrictions. Most of the platforms described are still in the research or prototype phase, with limited commercial availability, incomplete automation, and insufficient interlaboratory validation in complex food matrices. Furthermore, many systems still face challenges related to receiver cost, batch-to-batch reproducibility, long-term stability, and matrix effects, which reduce their robustness under routine analytical conditions. Most importantly, regulatory acceptance remains low compared to established chromatographic reference methods due to a lack of standardised protocols, harmonised validation frameworks, and formal demonstration of their suitability for official control. Therefore, these emerging sensor technologies should currently be considered as promising complementary or screening tools, especially for industrial or field applications, rather than as direct replacements for reference methods validated in official control environments.

**Table 6:** Summary of the conditions of different sensor models applied to histamine determination and performance parameters.

Detection Strategy	Fundamentals	Matrix	Sample Treatment		Precision (% RSD)	Range (Method)	Linearity (R^2^)	Recovery (%)	Recognition Element	LOD	LOQ	Ref.
			Preparation	Range (Sample)								
OPTICAL												
Fluorescence	Biosensor based on specific binding bioreceptors (peptides) and CQD	Mackerel	TCA extraction	1.64–573.91 mg/kg	0.71–11.15	1–100 mg/L	0.974	88.17–109.72%	NAC-CQDs/Pep (biosensor)	0.013 mg/kg	0.039 mg/kg	[91]
Fluorescence	Sensor based on UiO-66 ZrOFs functionalised with hydroxyl groups and partial replacement of Zr(IV) with Terbium (III)	White shrimp	No extraction needed	4.85–40.51 mg/kg	5 (1.3–3.3)–10 (2.1–2.7)–20 (1.5–3.2)–40 (1.8–2.1) mg/L	0–53.3 mg/L	0.991	5 mg/L: 97–107%; 10 mg/L: 99.40%; 20 mg/L: 90.55–107.45%; 40 mg/L: 90–101.28%	ZrOFs, and partial replacement with Terbium(III) ions	0.46 mg/L	1.38 mg/L	[88]
Fluorescence	Biosensor combined with magnetic separation based on the competitive principle of antibody–antigen specific recognition	Meat and fish products, cheese, soya sauce, rice vinegar & rice wine	TCA extraction. Purification with n-hexane	0.19–70.80 mg/kg	0.07–2.84	1 × 10^−5^–0.1 mg/L	0.9954	-	Upconversion nanoparticles: anti-HIS linked with NaYF4Yb, TM and NAYF4Yb, Er UCNP	1 × 10^−5^ mg/L	3 × 10^−5^ mg/L	[90]
Fluorescence	FRET system based on CD-modified nanoporous alumina membrane and Fe_3_O_4_@Au magnet nanocomposites	Mackerel	TCA extraction. Purification with 1-pentanol + HCl	10.00–389.03 mg/L	-	1.11 × 10^−5^–1111.5 mg/L	0.9985	-	CD-modified nanoporous alumina membrane	7.78 × 10^−6^ mg/L	2.33 × 10^−5^ mg/L	[116]
Fluorescence	Sensor based on HB doped in NPS and surface modification with FC to provide the FRET On-Off process	Salmon and Tuna	PBS 0.05 M	27.01–62.13 mg/kg	0.24–5.92	3.24–18.53 mg/L	0.99	26.5 mg/L: 101.9–110.3%; 50.5 mg/L: 98.7–104.6%; 59.5 mg/L: 96.4–104.4%	Organic small molecules and organic polymers (HB@NPS@FC)	0.188 mg/kg	0.564 mg/kg	[117]
Fluorescence	Nanoclay-based turn on mode of the Eu^3+^ complex nanocomposite with HIS response	Raw fish	Direct	0–30 mg/kg	-	2–14 mg/L	0.99317	-	Eu(DBM)@nanoclay	0.935 mg/L	2.805 mg/L	[118]
Fluorescence	Sensor generating dual stimulus-response fluorescence based on dye covalently modified EuMOFS	Reference material	Direct	-	-	0–27.79 mg/L	0.99028	-	Methyl red@lanthanide metal–organic frameworks (MR@EuMOFs)	0.0111 mg/L	0.0333 mg/L	[93]
Fluorescence	Sensor based on AIE (aggregation-induced emission) effect of DPA-CuNPs synthesised by a one-pot method	Fish, pork, red wine	TCA extraction Purification with n-hexane and dried	2.22 × 10^−3^–0.440 mg/L	0 (0.01)–0.445 (0.10–0.13) mg/L	5.56 × 10^−3^–0.556 mg/L	0.9963	0.445 mg/L: 91.3–99.1%	DPA-CuNPs	3.33 × 10^−3^ mg/L	0.01 mg/L	[119]
Fluorescence	Dual-emission ratiometric fluorescent sensor using filter paper with pH-responsive carbon dots	Fresh shrimp & mushroom	Direct	Qualitative	1.4–6	0–8.34 mg/L	>0.9944	-	Nitrogen and sulphur-doped carbon dots (N,S-CDs) & Rhodamine B	0.119 mg/L	0.357 mg/L	[92]
Fluorescence	Photoluminescence sensor based on thioglycolic acid-modified CdTe QDs	Tuna, wine and dairy products	SPME (cationic solid phase extraction)	99.9–1687.3 mg/kg	17.78 (2.3); 46.68 (3.1) mg/L	1.1–63 mg/L	0.998	72 mg/kg: 106.2–108.2%; 220 mg/kg: 99.9–103.8%	Thioglycolic acid-modified CdTe quantum dots	1.1 mg/L	3.3 mg/L	[120]
Fluorescence	Photoluminescence sensor based on copper nanoclusters	Fish, shrimp & red wine	TCA extraction Purification with n-hexane and dried	5.57–17.18 mg/L	0.11–0.43	0.0111–1.111 mg/L	0.9981	5.56 mg/L: 100.2–101.2%; 11.2 mg/L: 100.3–101.7%; 16.67 mg/L: 101–103.1%	CuNCs	6.67 × 10^−3^ mg/L	2 × 10^−2^ mg/L	[121]
Fluorescence	Biosensor based on a competitive pseudo-immunoassay using molecularly imprinted polymers	Canned tuna	Chloroform extraction, evaporation/purification with hexane	0.111–47.79 mg/L	-	-	-	-	Organic small molecules and organic polymers: competitive fluorescent MIP methacrylic acid as a functional monomer	0.02 mg/L	0.06 mg/L	[122]
Fluorescence	Dual mode fluorescence sensor based on aptamer recognition and FRET	Fish	-	8.34 × 10^−3^–0.105 mg/L	0.0222 (3.4)–0.0445 (2.7)–0.0667 (3.8)–0.0889 (1.4)–0.111 (2.6) mg/L	0.0222–0.111 mg/L	0.9992	0.0222 mg/L: 82.24–83.41%; 0.0445 mg/L: 92.87–97.41%; 0.0667 mg/L: 94.02–105.92%; 0.0889 mg/L: 94.97–101.94%; 0.111 mg/L: 91.88–94.98%	Aptamer and AuNPs	5.08 × 10^−4^ mg/L	1.68 × 10^−3^ mg/L	[99]
Colorimetric & Fluorescence	Colorimetric and ratiometric fluorescent sensor based on simple pH-sensitive aggregation-induced emission-active fluorogen (AIE-gen)	Shrimp & chicken	Direct	0.34–474.58 mg/kg	0.24–9.16	0.00348–0.0278 mg/L	0.984	-	4-((dimethylamino)styryl)quinoxaline-2(1*H*)-one (ASQ)	1.96 × 10^−3^ mg/L	5.89 × 10^−3^ mg/L	[123]
Colorimetric	Multicolorimetric biosensor based on that etched between the hydrogen peroxide generated by the reaction of HIS and DAO, and AuNRs	Bonito	TCA extraction	3.00–11.22 mg/L	0.78–3.70	0.020–0.1 mg/L	0.987	2.78 mg/L: 107.88%; 5.56 mg/L: 111.12%; 11.12 mg/L: 100.95%	Enzymatic reaction in gold nanorods (AuNRs)	1.485 mg/L	4.455 mg/L	[82]
Spectrophotometric	CHI/MIPs coated on Fe_3_O_4_ MNPs for selective SPE	Tuna fish	Ethanol extraction	0.0283–0.1403 mg/kg	0.04–0.1 (2.9–4.1) mg/L	0.005–0.16 mg/L	0.996	0.030 mg/L: 94.3–107%; 0.060 mg/L: 95.3–102%; 0.11 mg/L: 98.1–101.8%	Chitosan-based magnetic nanoparticles	1.5 × 10^−3^ mg/L	4.5 × 10^−3^ mg/L	[124]
SERS	Surface-Enhanced Raman Spectroscopy-based sensor using a silver colloid SERS substrate	Fish	1-butanol	-	-	0–200.07 mg/L	0.962	-	AgNPs	-	-	[67]
SERS	Molecularly imprinted polymers (MIPs) synthesised as artificial antibodies and SERS detection	Canned tuna	hexane	0–30 mg/kg	-	3–90 mg/L	0.947	-	AuNPs	-	-	[125]
ELECTROCHEMICAL												
Voltametric	MIECLS based on MXene and sodium ascorbate coordinated amplification CNNS signal strategy	White wine, white vinegar and fresh fish	TCA extraction. Purification for fat removal with n-butanol/trichloro-methane	4.23–209.23 mg/kg	5 (2.27–2.60)–10 (2.09–2.86)–25 (2.35–2.54)–50 (1.89)– 100 (2.24)–250 (4.51) mg/L	5.56 × 10^−4^–11.12 mg/L	0.9975	5 mg/L: 84.58–86.34%; 10 mg/L: 91.50–92.70%; 25 mg/L: 91.21–91.39%; 50 mg/L: 75.81%; 100 mg/L: 81.79%; 250 mg/L: 83.69%	Carbon nitride nanosheets	1.90 × 10^−4^ mg/L	5.7 × 10^−4^ mg/L	[104]
Voltametric	Solution-gated graphene transistor by using graphene, multiwalled carbon nanotubes and AuNPs	Tuna fish	Water extraction	0.0111–11.12 mg/L	3.33 (1.46) mg/L	0.333–11.12 mg/L	0.992	0.333: 90.15%; 1.11 mg/L: 90.93%; 3.33 mg/L: 104.93%; 11.12 mg/L: 120.48%	SGGT with Gra/MWNT/AuNP modified gate electrode	0.0111 mg/L	0.0333 mg/L	[115]
Potentiometric	Nano-molecularly imprinted polymer (nanoMIPs) in polyvinychloride membranes	Wine & fish samples	Acetate buffer 0.01 M pH 5	14.9–312.3 mg/kg	15 (5.75)–30 (4.3)–311.1 (5.08) mg/L	0.111–1111.5 mg/L		15 mg/L: 99.3%; 30 mg/L: 101.7%; 311.1 mg/L: 100.4%	nanoMIPs	0.124 mg/L	0.373 mg/L	[107]
Amperometric	Bienzymatic sensor using DAO and HRP immobilised into PS/carbon nanotubes/ferrocene membrane	Fish and seafood	Water extraction	23–208 mg/kg	**Intraday**: (5.6) **Interday**: (6.5)	0.0333–22.23 mg/L	0.9994	-	DAO & HRP co-immobilised into polysulfone/carbon nanotubes/ferrocene membrane	1.889 mg/L	5.668 mg/L	[108]
Amperometric	Chelating property between HIS and cupric (II) ions	Saury fish	Water extraction	7.56 mg/kg	0.6	0.111–83.36 mg/L	0.999	-	Copper electrode	0.0367 mg/L	0.110 mg/L	[126]
Impedimetric	Reduced graphene oxide platform	-	-	-	-	0.0111–0.111 mg/L	-	-	Reduced graphene oxide. Antigen–antibody binding events	0.0111 mg/L	0.0367 mg/L	[110]
Voltametric, Impedimetric & Amperometric	Electrochemical biosensor based on the SPC electrode modified with a CS-Au nanoparticles composite cryogel on PB-coated multiwalled carbon nanotubes	Fresh fish & shrimp	PBS 0.1 M pH 7.50	54.2–168.3 mg/kg	2.78 (0.4)–5.56 (1.2)–8.34 (0.9)–11.12 (1–2)–13.89 (0.5) mg/L	0.278–13.89 mg/L & 13.89–44.46 mg/L	0.999 & 0.992	2.78 mg/L: 209.6%; 5.56 mg/L: 199.6%; 8.34 mg/L: 185.9%; 11.12 mg/L: 176.2%; 13.89 mg/L: 163.7%	DAO-CS-AuNPs/PB/MWCNTs/SPCE: the electrode in the system is a screenprinted carbon electrode modified with PB-coated MWCNTs capped with a CS-AuNPs Cry layer entrapping DAO	0.201 mg/L	0.603 mg/L	[114]
Volatametric & Amperometric	Electrochemical enzyme biosensor	Sea bass	PBS pH 7.2	5.08–19.55 mg/L	8	1.111–111.15 mg/L	Co(II)-phthalocyanine: 0.998; Prussian Blue: 0.995	97.3–101.5%	DAO using magnetic beads as immobilisation supports on Co(II)-phthalocyanine/carbon and PB/carbon electrodes	Co(II)-phthalocyanine: 0.570 mg/L; & Prussian Blue: 0.533 mg/L	Co(II)-phthalocyanine: 5.497 mg/L; & Prussian Blue: 4.236 mg/L	[120]
Voltametric & Amperometric	Electrochemical Bio(immune)sensor coated w/O_2_ plasma-treated SWCNTs	Fish	Water extraction	4.91 × 10^−4^–4.8 × 10^−2^ mg/L	3.2–8.5	5 × 10^−6^–5 × 10^−2^ mg/L	0.9805	96–104.7%	Ag electrode coated with single-walled carbon nanotubes (SWCNTs)	2.48 × 10^−6^ mg/L	7.44 × 10^−6^ mg/L	[113]
Piezoelectric	Electrochemical quartz crystal microbalance biosensor based on Sol–Gel MIP	Spiked fish products	Soxhlet	79.8–494.6 mg/kg	0.3–4.4	0.11 × 10^−2^–4.45 × 10^−2^ mg/L	0.9965	93.2–100.4%	MIP	7.49 × 10^−4^ mg/kg	2.47 × 10^−3^ mg/kg	[112]

### 3.6. Future Perspectives

Most of the histamine analysis methods for fishery products described in this article offer LOD/LOQ values below current regulatory thresholds; consequently, analytical sensitivity is generally not the primary obstacle to their use in official quality control. However, for regulatory purposes, the most critical criteria involve demonstrating accurate and precise quantification of the histamine levels specified in official control sampling plans, validating the method against reference methods, and ensuring that performance parameters meet the verification and uncertainty requirements mandated for laboratories accredited under ISO 17025:2017 [127]. In this context, standardised or equivalently validated HPLC methods remain the most suitable tools for official control; meanwhile, ELISA techniques, enzymatic assays, and novel sensor platforms are emerging as rapid screening tools that require chromatographic confirmation before control and compliance measures are implemented.

In this context, future histamine control is likely to evolve from isolated laboratory measurements toward interconnected, real-time systems that combine portable sensors, artificial intelligence, and digital traceability. AI-assisted interpretation is particularly relevant for histamine and other biogenic amines because machine learning can extract patterns from complex sensor outputs, support multimodal data fusion, and facilitate integration into HACCP-based decision systems. Smartphone-integrated sensing is also emerging as a practical application for histamine mobile-screening, with recent studies demonstrating histamine detection through smartphone-linked colorimetric, fluorescence, impedance, and electrochemiluminescence platforms in food products. Beyond individual devices, Industry 5.0 concepts suggest that histamine control could increasingly be embedded within smart seafood processing, where IoT sensors, cloud-connected laboratories, blockchain-enabled provenance, digital twins, and predictive analytics support adaptive quality management across the value chain. This transition appears especially relevant for fish products, where smart sensors, computer vision, electronic noses, and miniaturised spectroscopic systems are being developed for robust industrial environments and real-time monitoring from farm to fork. However, these advances will require standardised datasets, explainable models, stronger validation, improved portability, lower implementation costs, and clearer regulatory frameworks before widespread routine adoption becomes realistic, particularly for the fish industry and official control settings.

## 4. Conclusions

In this study, the current analytical methods and recent advances for HIS analysis in fish and fishery products were reviewed. Chromatographic techniques are widely employed to analyse HIS in food and biological samples. Of them, HPLC is the most common separation principle because it presents good accuracy, sensitivity, and reliability values, but presents several disadvantages, such as complex sample preparation, post-column derivatisation, and the use of high-quality organic solvents. To overcome these limitations, spectroscopic and enzymatic methodologies have successfully been applied to HIS analysis. Enzymatic methods, such as ELISA tests, have been developed and modified to obtain good correlations with HPLC methods. Nowadays, commercial enzymatic tests are widely used for being accurate, highly automated, and fast, and for avoiding the use of organic solvents. Recently, sensors based on optical, electrochemical, and/or biological nanomaterials were considered to be the most promising techniques applied to HIS analysis. The integration of nanomaterials, advanced fluorescent probes, and selective recognition elements such as aptamers, enzymes, and molecularly imprinted polymers (MIPs) has enhanced sensor performance, enabling histamine detection at low concentrations. Modern sensing platforms also benefit from miniaturisation, portable devices, and smartphone-assisted detection, which facilitate rapid and on-site analysis. These technological developments allow sensors to provide fast, reliable, and cost-effective monitoring of histamine in complex fish products, making them increasingly attractive alternatives to traditional laboratory-based analytical methods, and thus offering an excellent tool for the rapid and reliable detection of histamine throughout the food chain, which would contribute to improving food safety and consumer confidence. However, depending on the type of sensor, challenges like stability, interference, and lack of standardisation can still limit their ability to fully replace traditional laboratory methods.

## Figures and Tables

**Table 1:** Inclusion and exclusion criteria.

**Inclusion Criteria**	
Language	English
Year of publication	2015–2025
Type of document	Scientific articles (published in a peer-reviewed journal and systematic reviews)
Area of study	Food and Science Technology, Analytical Chemistry, Applied Chemistry, Biochemistry, Biotechnology, AND Nutrition
Title and abstract	Directly refer to the HIS analysis, AND matrix must be fish, OR fish product
Full text	Full-text papers with a focus on the method description, AND express HIS results independently, but as a set of BAs, AND/OR the description of the validation of the method, AND fish or fish products as a food matrix, AND the relevance of the study for the work objective
**Exclusion Criteria**	
Type of document	Clinic assays, books/book chapters, randomised controlled trials and conference proceedings
Study area	Microbiology AND Toxicology
Title and abstract	Words that do not refer to HIS, OR that refer to set of BAs where HIS is not included, AND information not relevant for the work objective
Full text	Articles that are not related to HIS analysis, OR that do not detail the analytical method followed for HIS analysis, AND articles of no relevance for the objective of the present work

## Data Availability

No new data were created or analysed in this study. Data sharing is not applicable to this article.

## Supplementary Materials

The following supporting information can be downloaded at: https://www.mdpi.com/article/10.3390/foods15152590/s1, PRISMA 2020 Main Checklist; Table S1. Extended description of chromatographic protocols.

## Abbreviations

The following abbreviations are used in this manuscript:

HIS	Histamine	IBCF	Isobutyl Chloroformate
BA	Biogenic amine	TLS	Thermal Lens Spectrometry
FDA	Food and Drug Administration	IC	Ionic Chromatography
HPLC	High Performance Liquid Chromatography	CD	Conductometric Detection
FL	Fluorescence	HS	headspace
DAD	Diode-array detection	FID	Flame Ionisation Detection
MS	Mass spectrometry	TLC	Thin-Layer chromatography
DNC-Cl	Dansyl chloride	LA-DBD	Laser Ablation Dielectric Barrier Discharge
GC	Gas chromatography	FAAS	Flame Atomic Absorption Spectroscopy
ELISA	Enzyme-Linked Immunosorbent Assay	UA-CPE	Ultrasonic-Assisted Cloud Point Extraction
MIP	Molecularly Imprinted Polymer	NIR	Near-Infrared Spectroscopy
LOD	Limit of Detection	SERS	Surface-Enhanced Raman Spectroscopy
LOQ	Limit of Quantification	IMS	Ion Mobility Spectroscopy
PRISMA	Preferred Reporting Items for Systematic Reviews and Meta-analyses	FT-MIR	Fourier Transform Mid-Infrared Reflectance Spectroscopy
TCA	Trichloroacetic acid	PLS-DA	Partial Least Squares-Discriminant Analysis
SPE	Solid-Phase Extraction	KNN	k-nearest neighbours
LSPTME	Liquid/Solid-Phase Transition Microextraction	SVM	Support Vector Machine
dLLME	Dispersive Liquid/Liquid Micro-Extraction	POCT	Point-of-care testing
MSPE	Magnetic Solid-Phase Extraction	ic-ELISA	Indirect competitive-ELISA
ACN	Acetonitrile	CLEIA	Chemiluminiscence Enzyme Immunoassay
DBS-Cl	Dabsyl chloride	MOFs	Molecular Organic Frameworks
FMOC-Cl	9-fluorenylmethoxycarbonyl chloride	NPs	Nanoparticles
NDA	naphthalene-2,3-dicarboxaldehyde	FRET	Fluorescence Resonance Energy Transfer
OPA	o-phtalaldehyde	LSPR	Localised Surface Plasmon Resonance
C18	octadecylsilane	CNNS	Carbon Nitride Nanosheets
C8	octylsilane	QCM	Quartz Crystal Microbalance
UPLC	Ultra-Performance Liquid Chromatography	SAW	Surface Acoustic Wave
HILIC	Hydrophilic Interaction Chromatography		
