# Supplementary material for: Current Analytical Methods and Recent Advances in Histamine Analysis in Fish and Fish Products: A Systematic Review
Source: Foods. 2026 Jul 23;15(15):2590. doi: 10.3390/foods15152590 (PMC13465368; doi:10.3390/foods15152590)
Supplement: Supplementary file 1 [file foods-15-02590-s001.zip › foods-4410059-supplementary-Table S1.pdf]

Table S1. Extended description of chromatographic protocols.

Analytical Technique	Matrix	Sample Treatment			Precision (%RSD)	Range (method)	Linearity (R^2)	Recovery (%)	HPLC parameters		LOD	LOQ	Ref.
		Derivatization	***Clean-up / Purification***	***Range (sample)***					**Column**	**HPLC conditions**			
**HPLC-UV**	Red wine and fish	DNS-Cl	dLLME	NR	**Intra-day**:<br>50 (3.1) - 200 (3.8) mg/kg<br>**Inter-day**:<br>50 (3.7) - 200 (3.1) mg/kg	10-1000 µg/kg	0.995	**Intra-day**:<br>50 mg/kg: 93.3%<br>200 mg/kg: 96.1%<br>**Inter-day**:<br>50 mg/kg: 94.5%<br>200 mg/kg: 98.9%	Shimadzu C18 (150 × 4.6 mm., 5 µm)	**Mobile phase:** Water:ACN<br>**Flow rate:** 1 mL/min<br>**Column Tª:** 30ºC<br>**Injection V:** 20 µL<br>**Detection:** 254 nm	2.8 µg/kg	9.6 µg/kg	Cao et al., 2019
**HPLC-UV**	Chinese mackerel fresh and canned products	DNS-Cl	Magnetic SPE ($CoFe_2O_4$/MEG)	16.1 - 56.1 mg/kg	2.76	1 - 50 mg/L	0.9999	**99.1%**	Agilent C18 (4.6 mm x 25 cm, 5 µm)	**Mobile phase:**<br>**Flow rate:** mL/min<br>**Column Tª:** ºC<br>**Injection V:** µL<br>**Detection:** nm	3.60 µg/kg	12 µg/kg	Guo et al., 2024
**HPLC-UV**	Chub mackerel fillets	Derivatisation was not required	NR	25.3 - 135 mg/kg	**Intraday:** 1.49 – 3.49<br>**Interday:** 1.63	0.15 - 12 mg/L	0.9998	42.5 mg/kg: 91.8% - 82.5 mg/kg: 87.5% - 165 mg/kg: 843%	SIELC Primesep 100 mixed-mode column (150 mm × 3.2 mm, 5-µm particles, 0.86 mL void volume)	**Mobile phase:** 10/90 ACN/water (A) and 50/50 ACN/water (B) acidified with 0.01 and 0.15% (v/v) TFA, respectively<br>**Flow rate: 0.5** mL/min<br>**Column Tª:** 30 ºC<br>**Injection V:** 20 µL<br>**Detection:** 210 and 267 nm	1.3 mg/kg	4 mg/kg	Zhao et al., 2025
**HPLC-UV**	Fish sauce	DNS-Cl	NR	1.88 - 24.4 mg/100g	**Intra-day**:<br>1 (9.1) - 5.2 (4.7) - 26 (0.7) mg/100g	0.12 - 120 µg/mL	0.9997	1 mg/100g: 85.8;<br>5.2 mg/100g: 88.5;<br>26 mg/100g: 87.6	Zorbax StableBond C8 (150 x 4.6 mm., 5 µm)	**Mobile phase:** phosphate buffer:ACN<br>**Flow rate:** 1 mL/min<br>**Column Tª:** 45ºC<br>**Injection V:** 10 µL<br>**Detection:** 254 nm	4 mg/kg	14 mg/kg	Zhong et al., 2023
**HPLC-UV**	Sausage and kielbasa	DBS-Cl	Electromembrane extraction microfluid chip-system	2.7 - 5.1 mg/kg	**Intra-day**:<br>Sausage – 120 (7.5) µg/L;<br>Kielbasa – 40 (6.9) µg/L	25 - 1800 µg/L	0.9902	Sausage:120 µg/L: 98.25%<br>Kielbasa:40 µg/L: 98%	ODS-3C18 (150 × 2 mm)	**Mobile phase:** 20 mmol/L acetate buffer containing 0.2% (v/v) triethylamine (pH = 6.0):ACN (10:90, v/v).<br>**Flow rate**: 1 mL/min<br>**Injection V:** 20µL<br>**Detection:** 440 nm	8 µg/kg	25 µg/kg	Zarghampour et al., 2018
**HPLC-UV**	Raw and canned tuna	DNS-Cl	NR	0.86 - 1279 mg/kg		5 - 1500 mg/L	0.9936		Spherisorb ODS (125 × 4 mm., 2.5 µm)	**Mobile phase:** ammonium acetate 0,1 M:ACN<br>**Flow rate:** 1 mL/min<br>**Detection:** 254 nm	3 µg/kg	10 µg/kg	Ghidini et al., 2021
**HPLC-UV**	Kutum (*Rutilus frisii*), common carp (*Cyprinus carpio*), and caspian salmon (*Salmon caspius*)	Benzoyl chloride	NR	0.22 - 168.36 mg/kg	**Intra-day**: (3.2); **Inter-day**: (4.4)	0.5 - 100 µg/mL	0.9991	**Intra-day**:<br>5 µg/mL: 94.5%;<br>20 µg/mL: 95.28%	Waters Symmetry C18 (150 x 3.91 mm., 5 µm)	**Mobile phase:** methanol:water<br>**Column Tª:** 38ºC<br>**Injection V:** 20 µL<br>**Detection:** 254 nm	40 µg/kg	160 µg/kg	Aflaki et al., 2016
**HPLC-UV**	Milkfish and Indian whiting	DNS-Cl	NR	17.28 - 28.69 mg / 100g		17.28 - 28.69 mg/100mL	0.903; 0.91	NR	LiChrospher 100 RP-18 reversed-phase (125 x 4.6 mm., 5 µm)	**Mobile phase:** ACN:water<br>**Flow rate:** 1 ml/min<br>**Detection:** 254 nm	NR	NR	Arulkumar et al., 2016
**HPLC-UV**	Tuna, sardine, kilka and mackerel	NR	NR	9.66 - 71.66 mg/kg	**Intra-day**: (1.3)	5 - 50 mg/L	0.9909	82%	C18 (250 x 4.6 mm., 5µm)	**Mobile phase:** 85% buffer solution:15% methanol: 15% ACN<br>**Flow rate:** 1 mL/min<br>**Injection V:** 20 µL<br>**Detection:** 214 nm.	1.5 mg/kg	5 mg/kg	Peivasteh-Roudsari et al., 2020
**HPLC-UV**	Canned fish	DNS-Cl	Carbon Spheres QuEChERS Extraction	12 - 282.5 mg/kg	**Intra-day**: (2.3)	1 - 50 mg/L	0.9999	97.7%	ZORBAX Eclipse XDB C18 (250 x 4.6 mm., 5 µm)	**Mobile phase:** 0.01 mol/L ammonium acetate with 0.1% acetic acid/ACN (1:9, v/v) + 0.01 mol/L ammonium acetate solution containing 0.1% acetic acid/ACN (9:1, v/v)<br>**Flow rate:** 0.8 mL/min<br>**Column Tª:** 30 ºC<br>**Injection volume:** 10 µL<br>**Detection:** 254 nm	7.2 µg/kg	97.7 µg/kg	Guo et al., 2022

**HPLC-UV**	Powdered parmesan cheese	Derivatization was not required	NR	NR	**Repeatability of peak height**: (1.49)	1.4 - 100 mg/L	0.999	NR	Chromolith monolithic silica-based cyanopropyl (30 x 4.6 mm)	NR	0.5 mg/kg	1.4 mg/kg	Gama & Rocha, 2020
**HPLC-UV**	Fish flesh	DNS-Cl	Toluene	24.01 - 200.30 mg/kg	**Intra-day**:<br>25 (18.2) - 100 (6.11) - 220 mg/kg (3.20);<br>**Inter-day**:<br>25 (32.23) - 100 (93.10) - 220 (91.05) mg/kg	25 - 100 – 220 mg/kg	NR	25 mg/kg: 96.03%;<br>100 mg/kg: 93.10%;<br>220 mg/kg: 91.05%	Kromasil C18 reverse phase (250 × 4.6 mm., 5 µm)	NR	NR	NR	Duflos et al., 2019
**Indirect RP-HPLC-UV**	Fish (*Parabramis pekinensis)*	NR	NR	NR	**Intra-day**:<br>1 (2) - 4 (1.7) - 3.62 (3.62) - 16 (0.83) mg/kg	1 - 32 mg/L	0.9995	1 mg/kg: 86%;<br>4 mg/kg: 87.25%;<br>8 mg/kg: 96.62%;<br>16 mg/kg: 86.62%	C18 (250 × 4.6 mm., 5 µm)	**Mobile phase**: 50% ACN:50% 2,5-dihydroxybenzoic acid 6mM/L pH 2.65<br>**Flow rate:** 0.8 mL/min<br>**Column Tª:** 35ºC<br>**Injection V:** 20 µL<br>**Detection:** 205 nm	1 mg/kg	3 mg/kg	Nadeem et al., 2019
**narrow-bore HPLC-UV**	Soy sauces, fish sauces, and dark malt beverages	Coumarin-3-carboxylic acid	n -hexane	1 - 368.8 µg/g	**Intra-day**:<br>10 (3.8) - 150 (3) -350 (5.3) µM;<br>**Inter-day**:<br>10 (5.2) - 150 (2.5) - 350 (1.6) µM	3 - 400 µM	0.992	10 µM: - 2.8%;<br>150 µM: 2.1%;<br>350 µM: 2.2%	ACQUITY UPLC BEH C18 (75 µm × 50 mm., 1.7 µm)	**Mobile phase**: 0.1% formic acid:ACN<br>**Flow rate:** 180 µL/min<br>**Injection V:** 1 µL<br>**Detection:** 294 nm	1 µM	3 µM	Tsai et al., 2021
**HPLC-UV**	Vinegar, baijiu, and chub mackerel	NR	NR	103 - 487 mg/kg	**Intra-day**:<br>2 (4.9) - 5 (4) µg/mL	0.1 - 10 µg/mL	0.9999	2 µg/mL: 98.2%;<br>5 µg/mL: 99.1%	Agilent Zorbax SB-C8 (150 × 4.6 mm., 5 µm)	**Mobile phase**: 50 mM KPF6 in 10 mM H_3PO_4 (pH 2.2):ACN<br>**Flow rate**: 1.5 mL/min<br>**Column Tª**: 30ºC<br>**Injection V**: 10 µL<br>**Detection:** operated at dual-wavelength mode, 210 nm for quantitation and a different wavelength for coelution check	10 µg/g	33 µg/g	Zhong et al., 2015
**HPLC-UV-DAD**	Fish products	DNS-Cl	NR	<3 - >720 mg/kg	**Intra-day**:<br>122 (1.6) - 233 (2.1) - 375 (2.1) mg/kg;<br>**Intermediate precision**: (2.7)	0 - 720 mg/L	0.9998	122 mg/kg: 100%;<br>233 mg/kg: 97%;<br>375 mg/kg: 103%	Zorbax Eclipse XDB C18 (150 x 4.6 mm., 5 µm)	**Mobile phase:** Ammonium acetate 0.1 M pH 7.9 :ACN<br>**Column Tª:** 40ºC<br>**Flow rate:** 1 mL/min<br>**Injection V:** 20 µL<br>**Detection:** 254 nm	3 mg/kg	10 mg/kg	Altieri et al., 2016
**HPLC-DAD**	Anchovies	DNS-Cl	NR	0.27 - 51.0 mg/kg	NR	NR	0.9980		Zorbax Eclipse Rapid Resolution XBD C18 (50 x 4.6 mm, 1.8 µm)	**Mobile phase:** methanol/water 75/25<br>**Flow rate:** 1 mL/min<br>**Column Tª:** 25 ºC<br>**Injection V:** 20 µL<br>**Detection:** 254 nm	0.06 mg/kg	0.20 mg/kg	D'Anza et al., 2024
**HPLC-UV**<br>**HPLC-FL**	Canned fish, sausages, beef (veal), and meat-based and tuna-based pet food	DNS-Cl	Salting-out-assisted liquid-liquid extraction	2.61 - 36.7 mg/kg	UV-**Intra-day**: (2.3);<br>**Inter-day**: (9.4)<br>FD-**Intra-day:** (4); **Inter-day:** (10)	1 - 10 mg/L	UV: 0.998;<br>FD: 0.9993	UV: 93.6%;<br>FD: 111%	Phenomenex Gemini C18 (150 x 4.60 mm., 3 µm)	**Mobile phase:** ACN:0.01 M ammonium acetate<br>**Flow rate:** 0.5 mL/min<br>**Injection V:** 20 µL<br>**Detection:** UV – 254 nm; FD – 320 nm (excitation), 523 nm (emission)	UV: 0.17;<br>FD: 1.6<br>(mg/kg)	UV: 0.5;<br>FD: 4.9<br>(mg/kg)	Francisco et al., 2019
**HPLC-FL**	Canned mackerel	OPA	SPE	UV: 10.59 - 23.44 mg/kg;<br>FLD: 6.67 - 25.10 mg/kg	**Intra-day:**<br>12.5 (1.41) mg/kg;<br>25 (2.24) mg/kg;<br>50 (1.63) mg/kg;<br>100 (1.37) mg/kg; 200 (1.38) mg/kg	12.5 - 200 mg/L	0.999981	12.5 mg/kg: 98.49%;<br>25 mg/kg: 98.60%;<br>50 mg/kg: 98.72%;<br>100 mg/kg: 99.02%;<br>200 mg/kg: 98.88%	Thermo scientific ODS Hypersil C18 (250 x 4.6 mm., 5 µm)	**Mobile phase:** K_2HPO_4 20mM (adjusted to pH 4 with phosphoric acid):ACN<br>**Flow rate:** 0.9 mL/min<br>**Column Tª:** 30ºC<br>**Injection V:** 30 µL<br>**Detection:** 345 nm (ex), 445 nm (em)	1.8 mg/kg	UV: 8mg/kg;<br>FLD: 5mg/kg	Kounnoun et al., 2020b

**HPLC-FL**	Fish products	FMOC-Cl	NR	0.03 - 1.16 µg/kg	**Intra-day:** (1.73); **Inter-day:** (7.38)	0.16 - 5 µg/mL	0.9998	103%	Waters 2475 C18 (150 × 2.1 mm., 3 µm)	**Mobile phase:** ACN:deionized water (63:37, v/v)<br>**Flow rate:** 0.34 mL/min<br>**Injection V:** 0.5 µL<br>**Detection:** 267 nm (ex), 314 nm (em)	0.10 µg/mg	0.30 µg/mg	Munir et al., 2021a
**HPLC-FL**	Fresh tuna	OPA	NR	2.06 - 83.73 mg/kg	**Intra-day**:<br>10 (2.3) µg/mL;<br>20 (2.6) µg/mL;<br>30 (2.3) µg/mL;<br>**Inter-day**:<br>10 (6.1) µg/mL;<br>20 (6.2) µg/mL;<br>30 (5) µg/mL	0.2 - 50 µg/mL	0.9996	80 – 110%	Nova-Pak C18 (300 x 3.9 mm., 4 µm)	**Mobile phase:** 0.2 mol/L sodium acetate:15 mmol/L 1-octane-sulfonic acid sodium salt solution adjusted to pH 4.9 with acetic acid + ACN<br>**Flow rate:** 0.5 mL/min.<br>**Column** and the **post column**: 22ºC. **Detection:** 340 nm (ex), 450 nm (em)	0.03 mg/kg	0.09 mg/kg	Evangelista et al., 2016
**Column-Switching HPLC-FL**	Fish meat and fermented foods	DNS-Cl	diethyl ether	2.89 - 22368.18 mg/kg		0.81 - 809.69 nmol/mL	0.9996	1.563 µmol/mL: 68.54%;<br>3.125 µmol/mL: 83.78%;<br>6.25 µmol/mL: 83.04%	(1) Develosil XG-C30M-5 (75 × 4.6 mm., 5µm) (2) Develosil XG-C30M-5 (150 × 4.6 mm., 5 µm)	**Mobile phase:** ACN:water, 65/35% (v/v) and ACN:water, 75:25 (v/v)<br>**Flow rate:** 1 mL/min<br>**Injection V:** 10 µL<br>**Column Tª:** 40ºC<br>**Detection**: 325 nm (ex), 470 nm (em)	0.1666 nmol/mg	0.5553 nmol/mg	Ishimaru et al., 2018
**UPLC-FL**	Fermented food and fresh fish	OPA	NR	0.9 - 164.4 mg/kg	**Intra-day:** 100 (4.5) mg/kg	0.1 - 10 mg/L	Beyond methods (cITP-CZE & UPLC): 0.996	NR	Nova-Pak C18 (150 x 4.6 mm., 4 µm)	**Mobile phase:** 100 mM sodium acetate buffer (pH 4.5) + 5 mM sodium octane sulphonate and 150 mM sodium acetate buffer (pH 4.5) + 5mM sodium octane sulphonate + 75% (v/v) ACN<br>**Flow rate:** 1 mL/min.<br>**Detection:** 330 nm (ex), 460 nm (em)		1 mg/kg	Kvasnička et al., 2019
**UHPLC-FL**	Yellowfin tuna (*Thunnus albacares*)	OPA	NR	9.8 - 54.1148 µg/mg	**Intra-day**: (6.5); **Inter-day**: (6.94)	10 - 60 µg/mL	0.9999	10 µg/mL: 97.52%;<br>30 µg/mL: 89.68%;<br>60 µg/mL: 89.75%	C18 (250 x 4.6 mm., 5 µm)	NR	2.4 µg/g	8 µg/g	Perelonia et al., 2019
**UHPLC-FL**	Tuna	DNS-Cl	NR	150 - 625 mg/kg	**Intra-day**:<br>25 (11.6) mg/kg;<br>250 (5.4) mg/kg;<br>750 (9.1) mg/kg;<br>**Inter-day**:<br>25 (14.2) mg/kg;<br>250 (7.9) mg/kg;<br>750 (8.8) mg/kg	25 - 750 mg/L	0.9887	25 mg/kg: 80%;<br>250 mg/kg: 95%;<br>750 mg/kg: 103%	(1) Acquity UPLC BEH C18 (50 × 2.1 mm., 1.7 µm) (2) Acquity UPLC CSH C18 (150 × 2.1 mm., 1.7 µm)	**Mobile phase**: 0.1% phosphoric acid:CAN:MeOH<br>**Flow rate:** 0.2 mL/min<br>**Column Tª:** 40ºC<br>**Injection V:** 2 µL<br>**Detection**: 300 nm (ex), 500 nm (em)	2.71 mg/kg	8.31 mg/kg	Pataca et al., 2021
**HPLC-NDA-FL & HPLC-DBS-TLS**	Wine	OPA, NDA, DBS-Cl	NR	0.08 - 0.09 mg/kg	NR	LOQs - 1.2 mg/L	0.985 - 0.997	93 - 112	(4 x 3 mm)	**Mobile phase:** sodium acetate 0.05 M:tetrahydrofuran (96:4, v/v) + methanol **Flow rate:** 1 mL/min.<br>**Injection V:** HPLC-FL – 10 µL; HPLC-TLS – 8 µL<br>**Detection**: HPLC-FL - 356 nm (ex), 445 nm (em); HPLC-TLS - 413 nm	NDA-FL: 27; DBS-TLS: 11; DBS-DAD: 85 (µg/kg)	NDA-FL: 81; DBS-TL: 40; DBS-DAD: 282 (µg/kg)	Rivoira et al., 2017
**HPLC-DAD**	Cheese, fish, and fishery products and meat and meat products	DNS-Cl	NR	0.1 - 98.8 mg/kg	**Intra-day**: (2.6)	2.5 - 500 mg/L	1	**Intra-day**: 100/250/500 mg/kg: 99%	LiChrospher C18 analytical (250 x 4.0 mm., 5 µm)	**Mobile phase:** ACN:ammonium acetate 0,1 M<br>**Flow rate:** 1 mL/min<br>**Column Tª:** 40ºC<br>**Injection V:** 20 µL<br>**Detection:** 254 nm	0.1 mg/kg	0.3 mg/kg	Bogdanović et al., 2020
**HPLC-DAD**	*Salminus maxillosus, Scomber scombrus* and *Seriola dumerili*	DNS-Cl	NR	0.36 - 52.62 µg/g	NR	0.05 - 20 µg/mL	0.9999	NR	AcclaimTM 120 C18 (150 × 4.6 mm., 5 µm)	**Mobile phase:** ACN:Water (73:27).<br>**Flow rate**: 2 mL/min<br>**Column Tª:** 30ºC<br>**Detection:** 190–400 nm range	0.30 µg/mg	0.91 µg/mg	Pinto et al., 2016

**HPLC-DAD**	Probiotic yoghurts	Benzoyl chloride	n-butanol & diethyl ether	16.98 - 43.41 mg/kg	**Intra-day**: (1.06 - 15.74); **Inter-day**: (1.11 - 19.29)	1 - 50;<br>3 - 50;<br>5 – 50 mg/L	0.980 - 0.997	NR	Kromasil C18 (250 x 4.6 mm., 5 µm)	**Mobile phase**: ACN:water<br>**Column Tª**: 40ºC<br>**Injection V:** 50 µL<br>**Detection**: 254 nm	0.18 - 4.01 mg/kg	1 - 5 mg/kg	Vieira et al., 2020
**LC-MS/MS**	Fish and fish products	Py-Tag	NR	2 - 367 mg/kg	**Intra-day**: LOQ (3.22); 50 (2.8) - 100 (2.34) mg/kg	lower conc range: 0.1-3 ng/mL; higher conc range: 3-40 ng/mL	>0.999	LOQ: 94.2%;<br>50 mg/kg: 96.2%;<br>100 mg/kg: 96.2%	(1) ACQUITY UPLC CSH C18 (150 × 2.1 mm., 1.7 µm) (2) ACQUITY UPLC CSH Phenyl-Hexyl (150 × 2.1 mm., 1.7 µm)	**Mobile phase:** 0.1% (v/v) formic acid in water:0.1% (v/v) formic acid in ACN<br>**Flow rate:** 0.5 mL/min.<br>**Column Tª**: 50ºC<br>**Injection V:** 2 µL	NR	2 mg/kg	Shiono et al., 2021
**LC-MS/MS**	Salted mackerel fillet	NR	Ion-pair SPE	<LOQ / nd	**Intra-day**:<br>2 (2.1) - 7.5 (1.5) mg/kg;<br>**Inter-day**:<br>2 (3.3) - 7.5 (3.7) mg/kg	0.1 - 12.5 mg/L	1	2.5 mg/kg: 117.6%;<br>5 mg/kg: 114.9%	Acquity UPLC BEH C18 (100 × 2.1 mm., 1.7 µm)	**Mobile phase:** 5 mM NFPA in water + 5 mM NFPA in ACN<br>**Gradient:** 5–20% B for 1 min and then 20–40% B for 11 min<br>**Flow rate:** 0.2 mL/min<br>**Column Tª:** 40ºC<br>**Injection V:** 2 µL	0.01 mg/kg	0.05 mg/kg	Ochi, 2019
**HPLC-MS/MS**	Whey powder	NR	NR	89 - 413 ng/g	**Intra-day**:<br>5 (4.4) - 10 (3.9) - 25 (3.8) ng/mL;<br>**Inter-day**:<br>5 (6.7) - 10 (5.9) - 25 (5.8) ng/mL	1.6 - 1000 ng/mL	0.9969	64%	Zorbax–SB–Aq C18 analytical	**Mobile phase:** ACN:water, 10:90 (%v/v); and ACN: water, 90:10 (v/v)<br>**Flow rate** 0.3 mL/min<br>**Colum Tª:** 40ºC	0.47 ng/mg	1.6 ng/mg	Mirzaei et al., 2022
**HPLC-MS/MS**	Aquatic products	NR	n-hexane	"Less than the USA and EU acceptable levels"	**Intra-day**: (2.42) **Inter-day**: (6.17)	20 - 2000 µg/L	0.9916	2.5 mg/kg: 76.2 - 111.3%;<br>5 mg/kg: 70.9 - 105.7%;<br>12.5 mg/kg: 75.5 - 102.3%	(1)Kinetex F5 (100 × 2.1 mm., 2.6 µm) (2) TSK gel Amide-80 (100 × 2.0 mm., 5 µm) (3) ZIC-cHILIC (150 × 2.1 mm., 3 µm) (4) ACQUITY HSS T3 (100 × 2.1 mm., 1.8 µm)	**Mobile phase:** 0.5% formic acid:ACN<br>**Flow rate:** 0.3 mL/min<br>**Column Tª:** 40ºC<br>**Injection V:** 10 µL	1 mg/kg	2.5 mg/kg	Zhang et al., 2021a
**HPLC-MS/MS**	Sefood, liquor, fermented meat, vegetable and dairy products	NR	n-hexane	0.22 - 33.14 mg/kg	**Intra-day**: (3.66) **Inter-day**: (9.74)	1 - 100 µg/L	0.9995	0.25 mg/kg: 78.7 - 114.3%;<br>0.5 mg/kg: 70.9 - 104.5%;<br>1.25 mg/kg: 75.5 - 111.2%	XBridge® HILIC Waters Technologies (150 × 2.1 mm., 3 µm)	**Mobile phase**: 10 mmol/L ammonium formate and 0.5% formic acid in water, 10 mmol/L ammonium formate and 0.5% formic acid in ACN<br>**Flow rate:** 0.3 mL/min<br>**Column Tª:** 35ºC<br>**Injection V:** 2 µL	0.1 mg/kg	0.25 mg/kg	Zhang et al., 2021b
**HPLC-MS/MS**	Canned and fresh tuna	Derivatisation was not required	NR	0.45 - 2.85 mg/kg	**Intraday:** 1 (2.0) – 50 (1.1) – 100 (3.3) mg/kg<br>**Interday:** 1 (1.0) – 50 (1.2) – 100 (3.5) mg/kg	0.0625 – 150 mg/kg	0.9948	1 mg/kg: 93.5%;<br>50 mg/kg: 113%;<br>100 mg/kg: 103%	Acquity UPLC BEH Amide (2.1 × 150 mm, 1.7 µm)	**Mobile phase:** 95/5% MeCN/water (A) and 70/30% water/MeCN. Buffer 10 mM ammonium formate, pH 3 (in both solutions)<br>**Flow rate: 0.5** mL/min<br>**I**njection **V:** 5 µL<br>**Detection:** ESI+ mode. Capillary voltage set +3.5 kV. Desolvation Tª was 350 ºC; Ion source Tª was maintained at 150 ºC. Cone gas flow was 25 L/h, Des-olvation gas flow rate set at 650 L/h	0.019 mg/kg	0.0625 mg/kg	Lioupi et al., 2024
**UPLC-MS/MS**	*Scomberomorus niphonius, Ctenopharyngodon idellus, Cyprinus carpi,* and *Larimichthys polyactis*	NR	NR	2.3 - 15.4 mg/kg	**Intra-day**: (0.12) **Inter-day**: (1.17)	0.4 - 3 mg/L	0.9979	50 mg/kg: 82.88%;<br>75 mg/kg: 92.96%;<br>100 mg/kg: 101.19%	Luna 3 µm HILIC 200 A (150 × 3.0 mm)	**Mobile phase:** 5mM ammonium acetate:methanol<br>**Flow rate:** 0.32 mL/min (A) and 0.08 mL/min (B)<br>**Column Tª:** 35ºC<br>**Injection V:** 5 µL	0.064 ng/mg	0.50 ng/mg	Zhang et al., 2019a

**UPLC-MS/MS**	Yellowfin tuna *(Thunnus albacares*)	NR	NR	0.3 - 332.24 mg/kg	**Intra-day**: (0.7-3); **Inter-day**: (3-4)	5 - 400 mg/L	>0.9995	98 – 103%	Acquity BEH HILIC (Waters) (100 x 1.9 mm., 1.7 µm)	**Mobile phase:** 10 mM ammonium formate in 95:5 ACN:water and 20 mM ammonium formate (pH 3) in ultrapure water.<br>**Flow rate:** 0.6 mL/min<br>**Injection V:** 5 µL	NR	1 mg/kg	Pais et al., 2022
**IC-MS/MS**	Fresh fish and processed fish products	NR	NR	0.3 - 226.9 mg/kg	**Intra-day**: 500 (10) ppb; 5000 (5) ppb	0.03 - 10 mg/L	0.9982	100%	IonPac CG 17 cation-exchange (50 × 4 mm)	**Mobile phase**: formic acid 2 M:water<br>**Flow rate:** mL/min<br>**Injection V:** 25 µL	0.02 ng/g	0.06 ng/g	Kočar et al., 2021b
**IC-CD**	Fresh and fruit juices, meat products, and smoked fish	NR	Carrez solutions	1.45 - 335 mg/kg	**Intra-day**: (0.56 - 1.97) **Inter-day**: (3.6)	0.5 - 5; 5-100 mg/L	0.9991; 0.9994	98%	MetroSep C 4–100/4.0	**Mobile phase:** 6 mM HNO_3<br>**Flow rate:** 1 mL/min<br>**Pressure**: 8.50 MPa<br>**Injection V**: 20 µL	0.071 mg/kg	1.63 mg/kg	Jastrzębska et al., 2015
**Cation exchange chroma-UV**	Canned and frozen fish and sauces	OPA	NR	<LOQ - 1370 mg/kg	**Intra-day**: (0.2); **Inter-day**: (4.6)	0.5 - 15 µmol/L	0.994	91.3 - 117.9%	MetroSep C4 (150 × 4 mm., 5 µm)	**Mobile phase**: 7 mmol/L nitric acid<br>**Flow rate**: 1 mL/min<br>**Column Tª**: 60ºC<br>**Injection V**: 20 µL<br>**Detection**: 360/440 nm.	0.17 µmol/kg	0.5 µmol/kg	Tsiasioti & Tzanavaras, 2021

NR: Not reported
